# Stakeholder perceptions on patient-centered care at primary health care level in rural eastern Uganda: A qualitative inquiry

**DOI:** 10.1371/journal.pone.0221649

**Published:** 2019-08-28

**Authors:** Everlyn Waweru, Nandini D. P. Sarkar, Freddie Ssengooba, Marc- Eric Gruénais, Jacqueline Broerse, Bart Criel

**Affiliations:** 1 Department of Public Health–Health Systems and Equity unit, Institute of Tropical Medicine, Antwerp, Belgium; 2 Department of Public Health–Quality of Care, Athena Institute, Faculty of Science, Vrije University, Amsterdam, Netherlands; 3 Faculty of Social Anthropology and Ethnology, University of Bordeaux, Bordeaux, France; 4 ISGlobal, Hospital Clinic—University of Barcelona, Barcelona, Spain; 5 Department of Health Policy Planning & Management, Makerere University College of Health Sciences, Kampala, Uganda; University of New South Wales, AUSTRALIA

## Abstract

**Background:**

Patient-centered care (PCC) offers opportunities for African health systems to improve quality of care. Nonetheless, PCC continually faces implementation challenges. In 2015, Uganda introduced PCC as a concept in their national quality improvement guidelines. In order to investigate whether and how this is implemented in practice, this study aims to identify relevant stakeholders’ views on the current quality of primary health care services and their understanding of PCC. This is an important step in understanding how the concept of PCC can be implemented in a resource constrained, sub-Saharan context like Uganda.

**Methods:**

This qualitative study was conducted in Uganda at national, district and facility level, with a focus on three public and three private health centres. Data collection consisted of in-depth interviews (n = 49); focus group discussions (n = 7); and feedback meetings (n = 14) across the four main categories of stakeholders identified: patients/communities, health workers, policy makers and academia. Interviews and discussions explored stakeholder perceptions on the interpersonal aspects of quality primary health care and meanings attached to the concept of PCC. A content analysis of Ugandan policy documents mentioning PCC was also conducted. Thematic content analysis was conducted using NVivo 11 to organize and analyze the data.

**Findings and conclusion:**

While Ugandan stakeholder groups have varying perceptions of PCC, they agree on the following: the need to involve patients in making decisions about their health, the key role of healthcare workers in that endeavor, and the importance of context in designing and implementing solutions. For that purpose, three avenues are recommended: Firstly, fora that include a wide range of stakeholders may offer a powerful opportunity to gain an inclusive vision on PCC in Uganda. Secondly, efforts need to be made to ensure that improved communication and information sharing–important components of PCC–translate to actual shared decision making. Lastly, the Ugandan health system needs to strengthen its engagement of the transformation from a community health worker system to a more comprehensive community health system. Cross-cutting the entire analysis, is the need to address, in a culturally-sensitive way, the many structural barriers in designing and implementing PCC policies. This is essential in ensuring the sustainable and effective implementation of PCC approaches in low- and middle-income contexts.

## Introduction

Evidence suggests that most health systems in sub-Saharan Africa still struggle to provide quality primary health care to all the members of their population [1]. Shortages of staff, unreliable disbursement of medicines, inadequate health financing and transitioning health information systems are persisting challenges that further deepen inequities in the quality of care provided across countries [2–5]. The changing burden of disease with increasing non-communicable chronic diseases means that health systems have to care for patients with complex health care needs. In addition to preventive, promotive and curative care, some patients will also require continuing chronic care management with links to the community and social services [6]. The increasing demand for accountability within the functions of the health system has also warranted the involvement of both providers and consumers in defining health goals and evaluating both technical and non-technical aspects of quality [2, 7, 8].

Patient-centered care (PCC) has been proposed as one of the approaches to improve the responsiveness of health systems by focusing on interpersonal, psychosocial and cultural aspects of health care [9, 10]. In this vein, incorporating user experience as a measure of quality of care is another recommended strategy [2, 11, 12]. There are many definitions of PCC. Most of them are centered on providing care that is *“respectful of*, *and responsive to*, *individual patient preferences*, *needs and values*, *and ensuring that patient values guide all clinical decisions”* [13]. This, however, can be further interpreted to include reorganization of health services around patients' needs and expectations for health care [2, 14, 15]. Barry et al. [14] state that PCC is *“about considering people’s desires*, *values*, *family situations*, *social circumstances and lifestyles; seeing the person as an individual*, *and working together to develop appropriate solutions”*. While some definitions emphasize that the patient should be the judge of PCC [13], caution is due in cases where patient health literacy is low and patient expectations may not match minimal expected standards of primary health care [3]. Consequently, PCC implies a culture that considers patients and/or caregivers as equal partners in their own health, alongside health professionals in planning, developing and monitoring care in accordance to expected health standards. While this is accepted in principle, it provides a cultural challenge in empowering patients to see themselves as equal partners [16]; a professional challenge in prompting health workers to involve patients in decision-making by taking into account their lived experiences as a resource [17]; and last but not least, a research challenge in measuring its implementation [18].

Evidence also suggests that a patient-centered focus of primary health care contributes to improvements in quality of care [2, 8, 19, 20]. Benefits include the following: positive effects on health worker satisfaction [21], better reported patient outcomes [22], better understanding of the psychological aspects of a patient's problems [23], improved patient satisfaction with the care experience [24], improved patient confidence regarding treatment compliance [25], and better integration of preventive and promotive care [9]. Reviews, however, caution that evidence on the impact of PCC on health status remains mixed [26–28].

PCC approaches have been implemented in several African countries, including Uganda, but they are usually designed and implemented by health providers and tend to focus on disease-specific approaches. Examples include decentralization of anti-retroviral treatment to community level [29, 30]; improving health worker communication as part of integrating mental health service delivery into primary health care systems [31]; working with community health workers (CHWs) to directly administer tuberculosis medication [32]; strengthening peer-patients’ capacities to offer psychosocial support to patients with chronic conditions [33]; and training health workers to manage complications during delivery [34]. A scoping literature review by De Man et al. [35] outlines the current state of PCC in sub-Saharan Africa. It recommends focusing on factors that shape health provider performance in their clinical interaction with patients, but also on structural and organizational features of the local health system in which this interaction takes place, and on the broader socio-economic environment in which health workers operate. While PCC offers an opportunity for African health systems to improve the quality of primary health care by engaging both providers and consumers of health services, difficulties are experienced in first translating evidence to policy, and finally, policy into practice.

In 2015, Uganda, a low-income country in sub-Saharan Africa, introduced PCC as a concept in their national quality improvement guidelines [36–38]. In order to investigate whether and how this is implemented in practice, this study aims to identify relevant stakeholders’ views on the current quality of primary health care services and their understanding of PCC. This is an important step in understanding how the concept of PCC can be implemented in a resource constrained, sub-Saharan context like Uganda.

## Methods

### Design

This is a qualitative study using document analysis, In-Depth Interviews (IDIs), Focus Group Discussions (FGDs) and feedback meetings with different stakeholders at national, district and health facility level in Uganda.

In exploring the understandings of PCC, we use a funnel approach to first conceptualize stakeholders’ perceptions of the quality of primary health care in Uganda. We use recent renditions of the Donabedian model [8] for health‐care improvement to classify stakeholders’ perceptions of quality of primary health care into the categories of “Structure,” “Process” and “Outcome” for health‐care quality improvement. Akachi and Kruk [2] provide more details of how to measure changes in the quality of care, and bring attention to the inclusion of user experience as a measure of outcomes in the assessment of quality. Using this refined model of quality of care, this study first explores stakeholder perceptions of what is required at the structural level to provide good quality patient-centered primary care. Secondly, we describe the processes of care that consider the patient-provider interpersonal relationship, using Hudon et al.’s [39] framework, which combines the various PCC domains as defined by Stewart et al. [40] and Mead and Bower [41]. These domains of PCC include exploring perceptions on health and the illness experience, understanding the person as a whole, finding common ground, enhancing the patient doctor relationship, and health promotion. We also incorporate Santana et al.’s [20] conceptual framework on how to practice *person*‐centered care, which *“emphasizes the structural domain*, *which relates to the health‐care system or context in which care is delivered*, *providing the foundation for PCC*, *and influencing the processes and outcomes of care”*.

**Fig 1:**
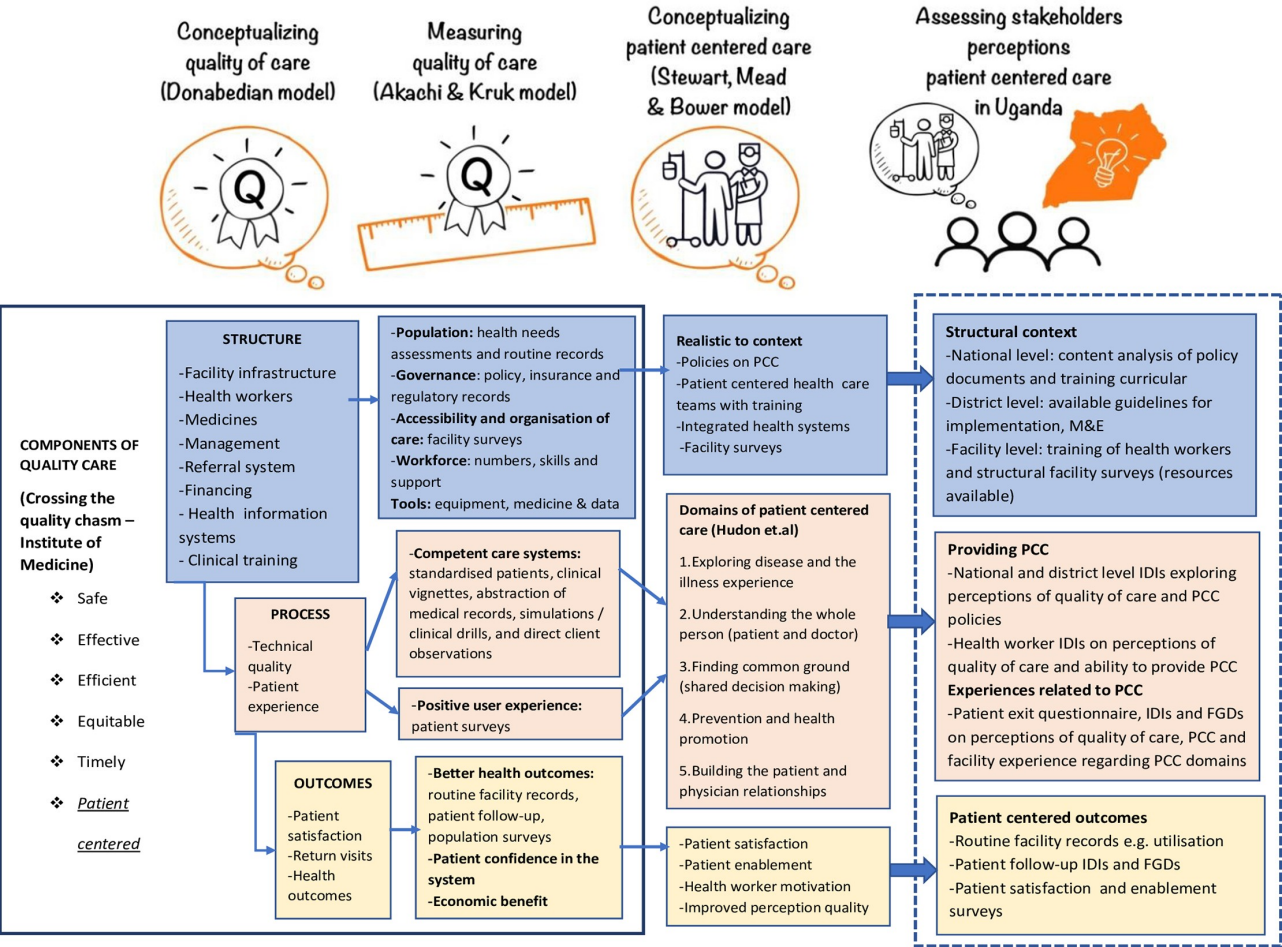
Combined frameworks used in this study for exploring stakeholder perceptions regarding quality and patient-centered care.

Data collection tools (including interview guides and questionnaires) were designed considering these two main areas of exploration: 1) Stakeholders’ perceptions of the quality of primary health care and contextual factors affecting its improvement; 2) Stakeholders’ understanding of PCC and factors affecting its implementation in the Ugandan context. As shown in Fig 1, this combination of frameworks is necessary in understanding stakeholders’ perceptions of PCC as an important part of improving the quality of primary health care that is provided in a context like Uganda.

### Setting

This study was conducted across multiple levels within the Ugandan health system. The Ugandan health system is organized into six levels of health care: level I comprises of village health teams (VHTs) and community medicine distributors; Health Centre II (HC II) led by an enrolled nurse; Health Centre III (HC III) led by a senior clinical officer; Health Centre IV (HC IV) and/or district hospital led by a senior medical officer; regional referral hospitals and finally the National Referral and Teaching Hospital (see illustration in supporting file S1 Fig).

At national level, we engaged and interviewed staff from the Ministry of Health, Makerere and Mbarara Universities, and NGOs involved in the provision of primary health care in Uganda. Most of the interviews at this level were conducted in Kampala. At the district level, this study was conducted in the eastern Ugandan districts of Iganga and Mayuge, within the Iganga Mayuge Health and Demographic Surveillance Site (IMHDSS). It has a population of about 67,000 people in about 13,000 households. The IMHDSS is located on the boundary between the districts of Iganga and Mayuge, about 115 km from the capital Kampala. The area is predominantly rural with only about 10% living in a peri-urban environment. The majority of people are of Busoga culture and speak the local language of Lusoga. The IMHDSS catchment area has one district hospital, four government HC IIIs, three non—government HC IIIs, five government HC IIs and three Non-Governmental Organization (NGO) HC IIs.

For the purpose of this study, we selected HC III facilities within the IMHDSS catchment area. A HC III has, on average, about 18 staff, led by a senior clinical officer, with a general outpatient clinic, a maternity ward and a laboratory. We purposively selected six HC III facilities according to the following criteria: inclusion of both governmental as well as private health facilities in both urban and rural settings; different demographic and epidemiological characteristics; different experiences with community strategy; and patient-centered approaches, if any. We hypothesize that patients with certain diseases (HIV/AIDS, tuberculosis (TB) or malaria), as well as maternal care patients, receive more patient centered care given the attention and additional resources available in funding, the additional training of staff, and evaluating outcomes of patients with these ‘programmatic’ diseases/conditions. This allowed us to analyze any differences in patients visiting the facilities for routine care, maternal and child health (MCH) or specialized clinics.

We selected three public HC IIIs (facility 1 and 2 in Iganga and facility 3 in Mayuge) and three private for-profit HC IIIs (facility 4, 5 and 6 in Iganga district). A short description of these facilities is presented in Table 1 below.

**Table 1:** Description of selected health centre IIIs.

	Location of facility	Type of facility	Facility in-charge interviewed	Services offered in addition to routine Outpatient Care, Maternal and Child Health Care, laboratory testing services and care for patients with HIV/AIDS (offered by all)	Link to community
**Facility 1**	Rural, Iganga district	Public HC III	1 clinical officer	Specialized care for patients with diabetes	2 VHT members, active
**Facility 2**	Semi-urban, Iganga district	Public HC III	1 clinical officer	No additional services	2 VHT members, inactive
**Facility 3**	Rural, Mayuge district	Public HC III	1 clinical officer	No additional services	1 VHT member, active
**Facility 4**	Semi-urban, Iganga district	Private HC III	1 facility administrator, 1 clinical officer	Inpatient care, dental care, ultra-sound and minor surgeries	5 social workers, active
**Facility 5**	Semi-urban, Iganga district	Private HC III (Faith -based)	1 clinical officer	Inpatient care and minor surgeries	3 social workers, active
**Facility 6**	Rural, Iganga district	Private HC III	1 midwife	This facility specialized in attending to pregnant women and young children	1 VHT member, active

### Stakeholder selection

First, we identified the different categories of stakeholders involved in the provision of primary health care in Uganda (a stakeholder is defined as any group or individual who can affect or is affected by the achievement of the organization's objectives [42, 43]). The stakeholders identified were grouped into nine categories as shown in Table 2. Next, several exploratory key informant interviews were conducted at the Makerere University School of Public Health to understand the organization of the Ugandan health system, select the district level setting, and identify relevant representatives of stakeholders from within the nine stakeholder categories.

**Table 2:** List and description of stakeholder categories.

	Stakeholder* category	Stakeholder description
Community level	1. Patients and caregivers	People who had visited the selected primary health care facilities for curative care. Caregivers are people who accompanied patients to the health facility and were interviewed on behalf of the patient.
	2. Village health teams	Community volunteers who are elected by their community members and are given basic training on major health programs so that they can mobilize and sensitize communities to actively participate in utilizing the available health services, provide health education and treatment of uncomplicated diseases, and contribute to community disease surveillance through active data collection and reporting–Ministry of Health guidelines [44].
	3. Local community	Local council members, patient support groups and families.
Health facility level	4. Health workers	Health care workers who directly provide health care to patients visiting primary health care facilities. These include the facility in-charges (clinical officers and nurses), midwives, laboratory technicians, pharmaceutical assistants, nursing aids and social workers.
District level	5. Health managers	Members of the District Health Management Teams and programme managers that are charged with the planning, implementation, monitoring and evaluation of health services at district and national level. They can be from the Ministry of Health or programme managers. Staff from the IMHDSS are in charge of “collecting routine data to provide a longitudinal data platform from more than 70,000 people to facilitate field trials for health, socio-economic assessment, agriculture and other technological interventions in rural and peri-urban populations”.
National level	6. Policy makers	Officials at the Ugandan Ministry of Health, Public Health Service Commission (in-charge of the health workforce), and regulatory bodies like the Uganda Nurses and Midwives Council and Allied Health Professionals Council. involved in policy-making to improve provision of primary health care in Uganda.
	7. NGOs	These are non-profit organizations that operate independently of the Ugandan government and have a role in the provision of primary health care. They could be focused on specific target patients or they could be cross-cutting.
	8. Academia	Academia include health educators and researchers. Health educators work in institutions that offer training to the health workers involved in the provision of primary health care, they come from both public universities and private nursing and clinical training schools.
	9. Development partners	These are bilateral or multilateral international partners who contribute resources for health including primary health care, for e.g. USAID, Marie Stopes.

*There were other stakeholders who were identified but we did not manage to engage with, including the Ugandan media that played a role during health worker strikes, as well as politicians who came in to get the health workers to end their strikes (see supporting file S2 Fig).

**Fig 2:**
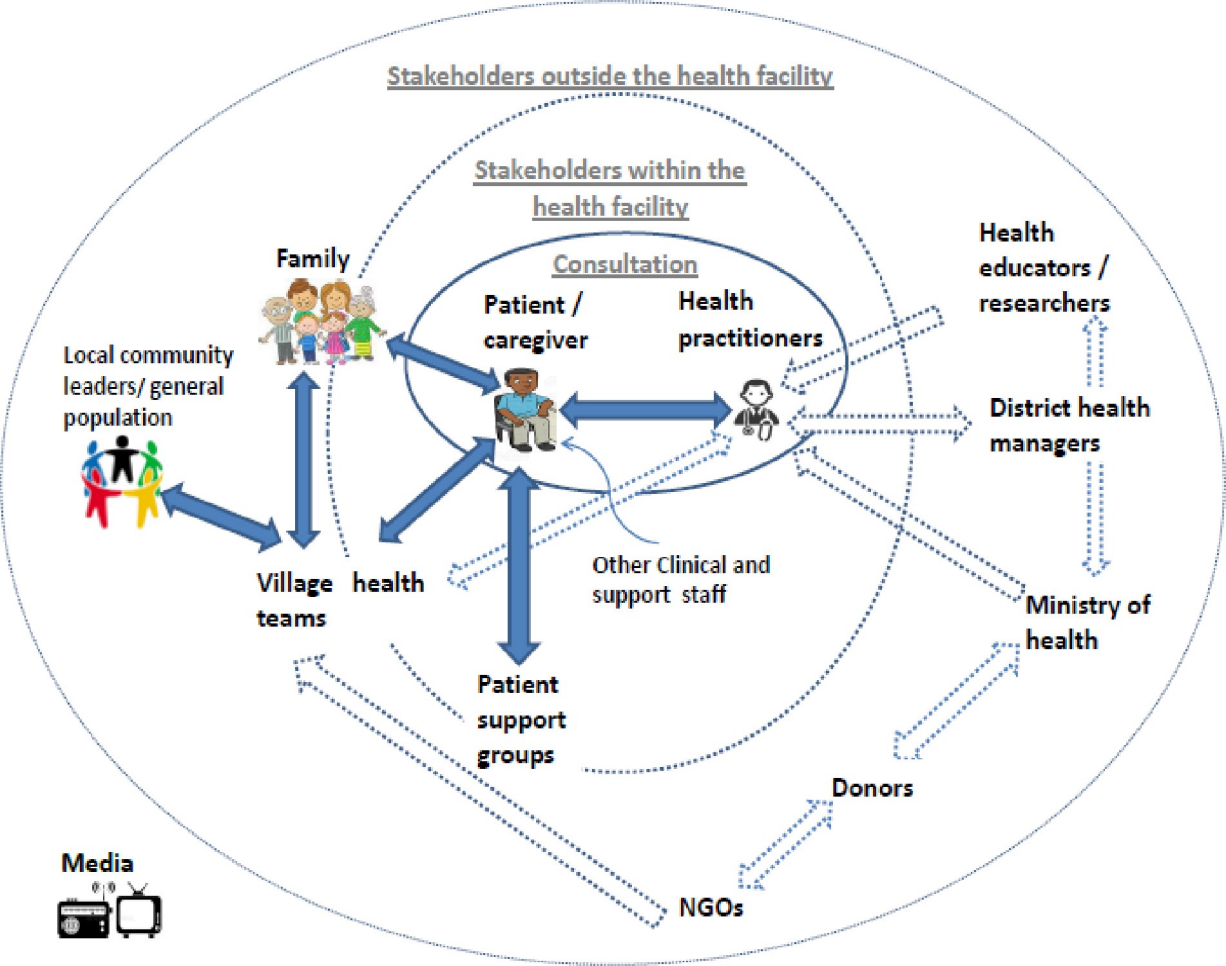
Mapping stakeholders involved in the provision of primary health care. A stakeholder map of actors involved in the provision of primary care based on document analysis, qualitative interviews and focus group discussions.

Fig 2 presents a stakeholder map with the key, identified Ugandan stakeholders who would be involved in the development, implementation and evaluation of strategies to improve quality of primary health care in general, and of PCC in particular. At the centre is the interaction between the patient/caregiver and the health worker within the context of a consultation. Within the primary health care facility, patients also interact (showcased by the dark blue arrows in Fig 2) with other clinical and support staff, other patients and sometimes VHT members, who then link patients and health workers to the general population/larger community.

While instituted nationally in 2001, VHTs only became active in Iganga district in 2010 [45, 46], with most VHTs supported by NGOs like The AIDS Support Organisation of Uganda (TASO) and research programmes (SMART 2D: Supporting patients with Type 2 diabetes in self-management). They interact directly with patients at the community level, and are supervised by the health workers at the facilities they are linked to and the program managers of NGOs working in their areas. Their roles are voluntary.

Other stakeholders related to the health system include district health managers, policy makers, researchers and academia who provide evidence and knowledge, NGOs and other donors who provide additional financial and non-financial support. Most stakeholders outside the health facility context do not directly interact with patients (reflected in Fig 2 by dotted lines). This points to the important role of the health worker as an initial advocate for the patient to health managers, policy-makers and donors. In order to provide PCC and ensure that patients are involved and contribute to discussions during consultations, alliances need to be formed at the health system level and beyond.

### Data collection

#### National level

In February 2018, IDIs were conducted with staff from the Ministry of Health, Makerere and Mbarara Universities, and NGOs. Interviews with policy makers and NGOs focused on understanding health care organization, legislative frameworks, health reforms/policies, resources available to primary health care facilities and any relevant PCC implementation initiatives in the Ugandan context. Interviews with academic participants explored whether aspects of quality of care, and more specifically PCC, were included in training curricula and student assessments. These interviews necessitated a narrative review of policy documents, news articles, and professional training guidelines that provide documented insight on the roles of potential stakeholders involved in the provision of primary health care in Uganda. A total of 11 policy documents were reviewed (see list in supporting file S1 Table).

#### District level

Following this, we engaged with the district health administrator who linked us with the District Health Management Teams (DHMTs) of Iganga and Mayuge districts, and the staff of the IMHDSS. After gaining permission to collect data in the primary health care facilities, we held two meetings at the district offices with members of the DHMT and staff of the IMHDSS once in February 2018 as an introductory meeting and again in August 2018 to discuss a summary of our findings.

#### Health facility level

At each of the six selected health facilities, an IDI was conducted with the facility in-charge or a health worker, at a time convenient to them. Interview topics included discussions on their roles in the provision of services, their perceptions about the quality of health care being provided at their facility, and their knowledge and perceptions of PCC including possible challenges and opportunities. Two feedback meetings were held with facility in-charges, once in February 2018 and again in August 2018 to discuss a summary of our findings.

#### Community level

Of the patients visiting the six HC IIIs during the study period, 37 patients were purposively recruited for in-depth interviewing at their homes. At each facility, two field assistants provided study information and gained initial consent from patients interested to participate. Only patients receiving curative care at the primary health care facilities were included in the study, in order to capture their perceptions of the process of care–i.e. the care that they received from the moment they entered the facility to when they left the facility. Furthermore, an effort was made to ensure that interviewees were representative of the three categories of patients receiving i) routine care, ii) curative maternal and child health care, or iii) attending a specialized clinic (people living with diabetes or HIV/AIDS).

If the patient consented, the principal investigator (PI), i.e. first author of the paper, would sit in during their consultation with the health worker. Following the consultation, a patient-exit questionnaire was subsequently filled in (in either the local language of Lusoga or English). The questionnaire collected quantitative data on patient demographics, and detailed patient experiences at the HC through Likert scale-based survey questions related to the 5 domains of PCC (exploring perceptions on health and the illness experience, understanding both the whole person, finding common ground, enhancing the patient doctor relationship, and health promotion) (see informed consent forms and questions asked in supporting file S1 Appendix). After the questionnaire completion, a request was made to schedule and later visit the patient at a time and place of their convenience for conduction of the IDIs.

**Fig 3:**
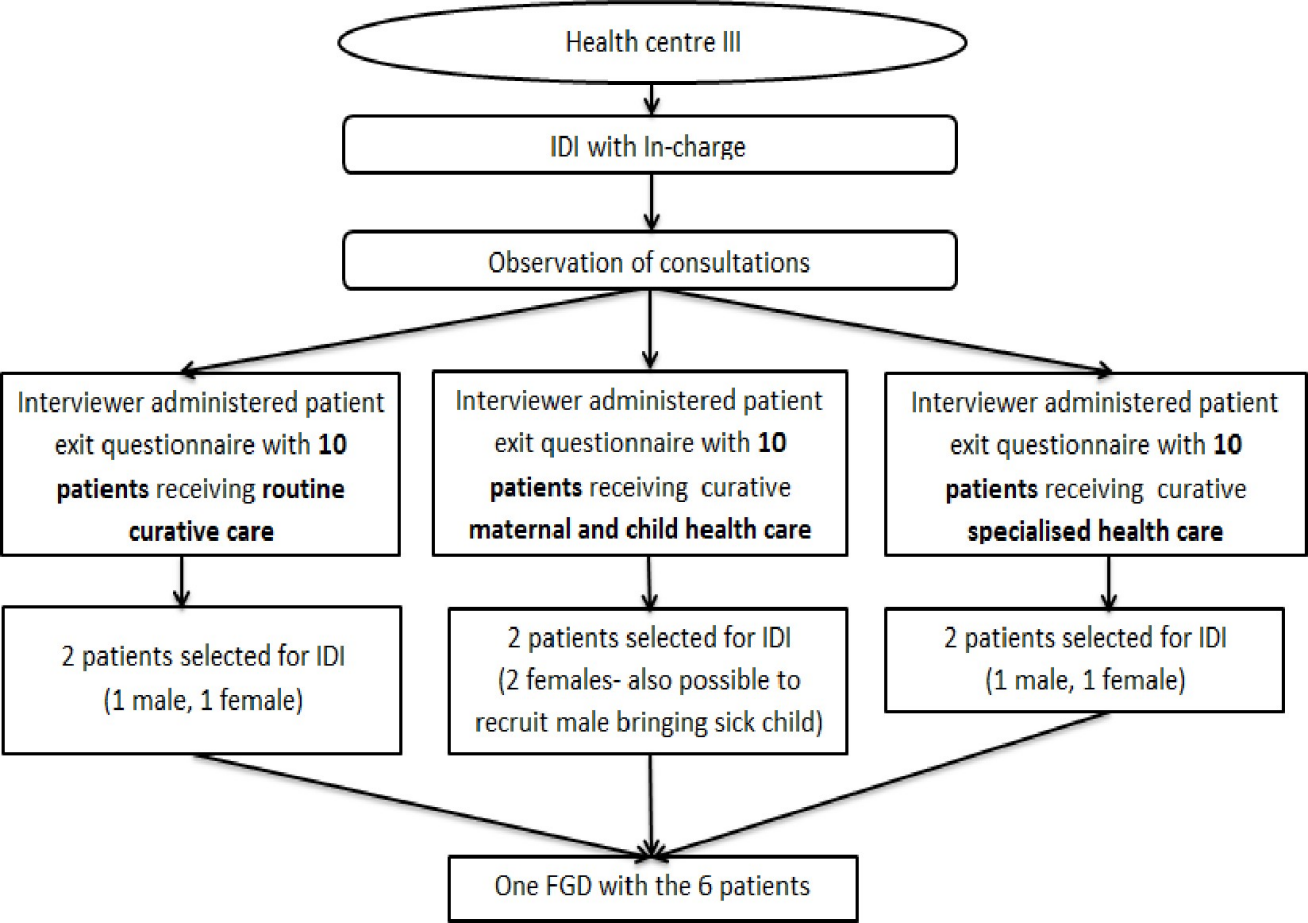
Selection of patients in each facility. A figure showing the process of selecting patients for exit interviews and follow-up in depth interviews and focus group discussions.

IDIs were conducted to solicit patients’ perceptions on health-seeking behavior, who they thought was responsible for their health, their experiences at health facilities, their relationships with health workers and VHTs, their membership in support groups, their awareness of their rights and responsibilities as a patient, as well as how all these factors contribute to their perceptions of the quality of primary health care available to them (see sample questions in supporting file S1 Appendix). A follow-up FGD was held with patients from each facility–the same as those who had participated in the IDIs–to validate and clarify the key messages. In total, 37 IDIs (facility 1 had an extra patient interviewed) and six FGDs were conducted with patients. Fig 3 delineates how patients were purposefully selected for IDIs and FGDs in each facility.

Additionally, a community mobiliser helped contact the VHTs and local council members serving in the catchment area of the six study facilities. Two discussions were held with VHTs and two discussions with local council members, respectively. Discussions were structured around the following: the perceived roles of VHTs and local council members, planning and execution of their daily activities, their motivation, and challenges they face, all in relation to primary health care provision.

In summary, a total of 49 IDIs, 7 FGDs, and 14 feedback meetings were conducted with stakeholders at facility, district and national levels between February 2018 and August 2018 (see Table 3; a sample of interview questions is included in supporting file S1 Appendix).

**Table 3:** Categories and numbers of respondents as per data collection method.

	Respondents	In-depth interviews	Focus group discussions	Feedback meetings
National level	Ministry of health, Quality Assurance department officers	**1**	_	**1** (10+ participants)
	Health Educators from Makerere and Mbarara Universities	**2**	_	
	NGOs: Regional Centre for Quality of Health Care; Uganda National Health Consumers Organisation	**2**	_	
District level	District health managers (DHMT and IMHDSS staff)	_	_	**2** (Introductory meeting—4 participants; Feedback / validation meeting—10+ participants)
Facility level (6 HC IIIs)	Health workers	**7** (4 clinical officers and 3 nurses)	**1** In-charges FGD (5 participants)	**1** (5 participants)
	Patients	**37**	**6** patient FGDs Participants in brackets (m-Male, f- Female): Facility 1 (4m, 3f); Facility 2 (2m, 4f); Facility 3 (2m, 3f); Facility 4 (3m, 3f); Facility 5 (4m, 2f); Facility 6 (1m, 5f)	**6** talks (with all patients present at facility during morning health talks)
	Community members (VHT and local council groups)	_	_	**4:** 2 with VHT (8 participants; 7 participants) 2 with local council members (3 participants in each)
Total*		**49**	**7**	**14**

*Data from structured questionnaires administered to patients and health workers are not included in this analysis

All national, district level and facility in-charge interviews and feedback meetings were conducted in English by the first author (PI of this study). IDIs with patients were conducted by the PI with a translator, while FGDs were conducted in Lusoga by two trained field assistants. Verbal or written consent was acquired from all respondents prior to all IDIs and FGDs. Information sheets were given to all respondents to retain in case they had future questions or complaints. All IDIs and FGDs were digitally recorded, while handwritten notes were made during all feedback meetings.

Ethical approva**l** for this study was obtained from the Institute of Tropical Medicine PhD committee (IRB/AB/ac/081), the Institute of Tropical Medicine Institutional Ethics Review Board (1166/17); University of Antwerp Ethics Review Board (17/24/278); the Makerere University School of Public Health Institutional Review Board (500); and the Ugandan National Council for Science and Technology. Prior to participation, written informed consent was obtained from stakeholders at national, district and facility level. In cases where patients could not write or provide a signature, verbal informed consent was obtained from patients and recorded in the study’s copy of the informed consent form.

### Data analysis

Data from all sources were converted into digital text format for analysis. Audio recordings of IDIs and FGDs were transcribed; data extracted from policy documents; notes from feedback sessions were typed using Microsoft word. Any text in Lusoga was translated into English. The written text was imported into NVivo 11, a qualitative data analysis software, for organization and qualitative thematic framework analysis as described by Gale et al. [47].

Thematic framework analysis of IDIs and FGDs was begun in field by the first author while conducting the IDIs and FGDs, as well as after the feedback meetings at facility, district and national level to validate themes and direction of analysis. Data analysis was categorized into two major areas of exploration: 1) Understanding stakeholder perceptions of the quality of primary health care and 2) Understanding stakeholders perceptions on the patient centered approach and factors that influence its implementation. Within each of these two major areas of exploration, we aimed to look at the structural context (resources available, policies in place, stakeholder perceptions of their roles); stakeholder perceptions of the processes involved in providing quality PCC; perceptions of their own roles in practicing PCC; and lastly, stakeholder experiences. Emerging themes from IDIs, FGDs, and feedback meetings were iteratively added and applied to the data as it was being collected. Post data collection, the research team developed a coding framework, guided by the PCC conceptual framework described previously (see Fig 1) as well as collected data. Data was coded according to the coding framework in NVivo 11 and organized into framework matrices or charts. Interpretation of data was conducted by the research team including the PI, research assistants who spoke the local language, and co-authors. The interpretation of data was also validated through feedback meetings with patients, health workers and district health managers.

Qualitative document analysis, as described by Glenn [48], was used to study the inclusion of PCC in the 11 Ugandan health policy documents that were analyzed. Each of the documents was investigated for whether improving quality of primary health care is included in the objectives; whether the policy specifically mentions the term PCC; whether the policy describes the conditions/resources needed for implementation of PCC; and finally, whether any indicators for monitoring the progress of implementation of PCC are proposed.

## Results

The quality of primary care is crucial to the achievement of patient-centered primary health care. Stakeholders are more likely to support patient-centered approaches if they are associated with improved quality and patient outcomes. This section will first describe stakeholder perceptions on the current quality of primary health care in the Ugandan context using Kruk et al.’s [2] quality of care model, and the role of other actors in this regard. This is followed by delineation of the existing Ugandan policies related to the improvement of the quality of primary health care in general, and patient-centered care (PCC) in particular. Finally, we present an analysis of stakeholder perceptions on PCC (using a combination of the Mead and Bower [41], and Stewart [40] models of PCC); and discuss factors that may enable or hinder the delivery of patient-centered primary health care in practice.

### Stakeholder perceptions on the quality of primary health care

In assessing the quality of primary health care, results are presented according to the framework of Kruk et al. [2]: (i) Structure (facility infrastructure, management and staffing); (ii) Process (technical [clinical] quality and patient experience); and (iii) Outcomes (patient satisfaction, return visits and health outcomes). Table 4 showcases related quotes from stakeholders on their perceptions towards the structural and procedural aspects of quality of primary health care.

**Table 4:** Quotes exemplifying structural and procedural aspects affecting the quality of primary care.

**Structural aspects of quality of care**	**Quotes**
Challenges with facility infrastructure, staff accommodation, and ambulances for referrals	*“At the health facility*, *there are also no spaces to sit*, *you need to squeeze yourself or sometimes sit on the floor”* (patient, female, 39 years, facility 1—public)
	*“Our staff have their own challenges*, *we don’t have accommodation near the facilities*, *so that is why most of the time they are late*, *they have to come from very far”* (facility in-charge, male, 55 years, facility 3—public)
	*“We share an external ambulance and sometimes you call the driver and he is somewhere else*, *this is a challenge especially at night”* (facility in-charge, male, 38 years, facility 5—private)
Availability of health workers	*“We have big challenges*, *…the number of staff is not enough and we have a very high workload… I also told you about our remuneration*, *it’s too little to cater for our needs*” (facility health worker, male, 29 years, facility 1—public)
Availability of medicines	*“You cannot have quality without resources… our resources are not enough*, *…sometimes some things that are crucial to the running of a facility are missing*, *when you order and you find an item is missing you have to wait until the next supply…two months later… so we are forced to refer complicated cases”* (facility in-charge, male, 51 years, facility 2—public)
	*“In other facilities you have to pay*, *here we get medication for free*, *but sometimes the medicine is finished and you have to come back to get it on another day”* (patient, female, 50 years, facility 2—public)
Autonomy in managing resources	*“In private sector*, *you have a lot of leverage to do things that are organized depending on what your team has decided but also you have the funds at your disposal to organize your delivery of health care in a way that is actually conducive for the patient but also for you as the health workers*. *But in the public*, *sector those things may not be very accessible and number one issue is funding*, *if you are going to procure something small you are going through a cascade of human beings and offices*, *that alone makes somebody lethargic to do”* (family medicine health educator, female, 54 years)
Health information systems	*“Every month we submit monthly reports to the districts but we never get feedback from them on how we are performing according to those reports”* (facility in-charge, male, 51 years, facility 2—public)
	*“… If a patient loses their book*, *it is still difficult to look for records from the last time they came… it would be easier if we had a computer but we need electricity first”* (facility in-charge, male, 55 years, facility 3—public)
Inadequate financing	*“The quality is not that bad and it is not that good*. *It is just average …with primary health care*, *we also look at disease prevention and health promotion on top of treating patients…but you find there is a challenge of funds*. *The money is never enough to fund all of the activities that need to be implemented*. *Secondly*, *even when it is not enough*, *it is not sent on time so we are not able to implement all the activities…not to our best”* (facility in-charge, female, 31 years, facility 1—public)
**Processes in the provision of care**	**Quotes**
Clinical training processes	*“Once they pass their clinical and written exams*, *they are out of our hands*, *they continue to their jobs… there is no way for us to know whether they are actually practicing what we trained them to do… resources are not all there*, *or the equipment to be used is different*, *and they have to then learn from the people who are there”* (family medicine health educator, female, 54 years)
	*“Every week we always have continuous medical education meetings where one of the doctors shares their experience with complicated conditions or changes in practice”* (facility administrator, female, 36 years, facility 4—private)
Supervision processes	*“We are supervised by the district*, *like once every three months… they try not to interfere with us while we are seeing patients but at the end we have a discussion and you know… its useful*, *its important*, *they help to keep us on track …sometimes we also tell them the challenges we are having and they report them back to the district”* (facility in-charge, female, 31 years, facility 1—public)
	*“The district supervisors rarely come here*, *we have to take the reports to them in the district… they should come here so that they really see how far it is and the condition that we are dealing with…”* (facility in-charge, male, 55 years, facility 3—public)
	*“We have a quality assurance team… who assess us every quarter*. *They are very strict*, *they even talk to the patients that they find here*, *complaints from patients are taken very seriously so we ensure that we always treat them well…”* (facility in-charge, male, 38 years, facility 5—private)
Referral processes	*“For us it is easy to refer because Iganga district hospital is just along the road*, *we try to follow up with the patient afterwards but it is not always possible*, *we cannot access the patients information or records of treatment from the hospital”* (facility in-charge, male, 38 years, facility 5—private)
	*“This facility is very far*, *patients walk for hours to reach us so when we refer them to Iganga district (hospital)*, *if they don’t have the means if is even more difficult for them to go there*, *so they stay at home and get very sick”* (facility in-charge, male, 55 years, facility 3—public)
Working with NGOs	*“We want to involve the community whether through contributing to buying lamps for night deliveries*, *or paying health workers little fees as a token when they have to come to the facility at night*, *or engaging traditional birth attendants in working together with health workers and mothers…it helps them to own the facility and the service…and gives normal people some power to converse with the health workers and voice their opinions…”* (NGO representative—UNHCO, male, 42 years)

Regarding structural aspects of primary health care, stakeholders agreed that substantial improvements had been made in increasing the number of health facilities. Nonetheless, health workers in rural public health facilities struggled with not having accommodation provided next to the facility which in turn affected their punctuality. Health workers from the public health facilities conveyed facing more structural challenges and were especially concerned about the inconsistencies in available resources including funding, human resources, medicines and salaries for health workers. Health workers in both public and private facilities insisted on the need for more ambulances to improve referral to the district hospital. Although health workers in private health facilities faced challenges in infrastructure, such as organizing referrals and patient follow-up, they were more confident about the general quality of care given at their facilities and appreciated the autonomy in planning and managing their resources (see Table 4).

Regarding the processes of quality primary health care, national level interviewees gauged technical (clinical) quality based on the continuous training of health workers, referral procedures and support supervision. The health workers themselves, from both public and private facilities, prioritized adequate compensation, continuous training and supervision as crucial to the provision of good clinical care. However, health workers in private facilities felt that they were subject to more strict supervision and viewed patients as evaluators, with consequences if patients complained. Health workers in both private and public facilities reported having periodic medical education sessions, but these were run by NGOs and confined to HIV/AIDS, malaria and tuberculosis. Health workers in one private facility mentioned internally organized monthly continuous medical education sessions. All the interviewed health workers expressed challenges in accessing patient information and following up patient care after they had referred patients to the higher level district hospital (see Table 4). Training and supervision paid little attention to the process of care from the patient’s perspective. Patient experiences were not documented or evaluated by health workers or their supervisors.

Regarding outcomes of care, patient experiences were perceived as satisfactory depending on the availability of medicines and polite health workers, easy access to the facility, time taken to get treatment, cleanliness of the facility and cost of care. Patients attending private health facilities seemed to be more satisfied with the cleanliness of the facility, 24-hour opening times, availability of medicines and ease of lab testing. Little emphasis was given to psychosocial aspects of care which will be discussed more in the section on PCC (and Table 5). Further data on patient satisfaction was collected through patient-exit questionnaires which will be published in a later article.

**Table 5:** Quotes exemplifying stakeholder perceptions on patient-centered care.

Element of PCC	Quotes
**Exploring perceptions of health and illness: *Responsibility for health***	*“The health worker is responsible for my health*. *He is the one to do the test*, *know what is wrong and give me medication to make me go back to normal”*(patient, male, 44 years, facility 2—public)
	*“I am the one who takes care of the home*, *I keep it clean*, *prepare the food and when my children are not feeling well*, *I am the first one to notice and tell my husband… he decides what we are going to do”* (patient, female, 29 years, facility 6—private)
	*“It depends on how serious the disease is*, *first we give Panadol*, *if it doesn’t resolve we go to the drug shop nearby*, *but when the condition is serious we just go to the health facility”* (patient, female, 29 years, facility 3—public)
Health seeking behavior	*“Some of the patients who come here relate their illness to traditional things… for some time now we give health talks in the morning teaching them about symptoms of common diseases… so now they come here instead of going to the abayiwa (traditional medicine men)… we used to have many deaths because of that but now it has reduced”* (facility in-charge, female, 31 years, facility 1—public)
**Understanding the patient as a whole person:** Discussing psychological and emotional issues	*“It is difficult to ask patients about everything in their lives*, *I only ask when it is related to their illness for example for patients with diabetes we advise them on diet and exercise and to make sure they take the medication everyday*” (health worker, male, 29 years, facility 1—public)
	*“There is a musawo (health worker) who I talk to when I have lady issues (sexual and reproductive health) or questions*, *she also closes the door… yesterday there was a man (male health worker)*, *and other people in the room… I will go again when the other musawo comes back… it is difficult to talk about these things with men in the room”* (patient, female, 33 years, facility 3—public)
**Understanding the health worker as a whole person**	*“We encourage the health workers working for us to be kind and consider the feelings of the patients*, *we take patient complaints very seriously”* (facility administrator, female, 36 years, facility 4—private)
	*“I see patients*, *manage the staff*, *order for drugs*, *write reports and look for funding… no one person can do all these things alone*, *so sometimes I get my colleague to see the patients and I handle the administrative things or take reports to the district”* (facility in-charge, female, 31 years, facility 1—public)
**Finding common ground—factors influencing communication and shared decision making** Health literacy	“Sometimes it can also depend on the literacy of the patient, there are those who cannot understand so much and only want to be treated and they don’t ask questions, those who ask questions we answer” (facility in-charge, female, 27 years, facility 6—private)
	“of us who are not very well informed, when we have questions, we are afraid to ask because we might disturb the musawo (health worker) but they write in the book and we go and ask the VHT what was written in the book” (patient, male, 28 years, facility 2 –public)
Information sharing	*“I always ask questions when I don’t understand*, *because I am paying for this care*, *if I don’t ask I will have to come back and pay again*! *The good thing is the health worker always answers and I understand what to do next”* (patient, male, 34 years, facility 4—private)
	*“I am satisfied*, *I did not understand why they did the test (blood test) but I got medication*, *and I feel better”* **(**patient, male, 44 years, facility 1—public)
	*“I always bring the book with me but I don’t know what is written in it*, *should I ask*?*”* (patient, female, 20 years, facility 2—public)
Awareness of patient rights	“We empower them to know about their rights and responsibilities …when the community is mobilized, they know which services they are supposed to get, like in a health centre III”(NGO representative–UNHCO, male, 42 years)
	“Because you know they (patients) don’t understand that it’s their right to get respectful care, they don’t understand that it’s their right to ask the provider that what exactly am I suffering from? How does it come about, is it anything I did wrong? …health workers do not communicate these things to them” (policy maker, male, 67 years)
**Factors affecting the doctor-patient relationship:** Professional values, training and assessment	*“We don’t just teach and examine the medical side*, *we look at—has this person been able to diagnose this… but also how are they talking to the patient*, *we also teach them about communication*, *we are teaching them to be observant*, *so that when they are treating patients they capture the whole picture not just treating a disease”* (nursing health educator, female, 52 years)
	*“You have to see the patient as the person*. *They are outside the facility*, *they are someone’s parent*, *child*, *teacher or student*, *you have to treat them with respect and consider where they come from*, *what is their faith and things like that… these are not things you are taught in school*, *you learn with experience”* (facility in-charge, female, 31 years, facility 1—public)
Respect, trust and empathy	*“We need to develop empathy*. *Suppose it was me who was coming to the facility and needed help*, *how would I want to be treated*?*”* (facility in-charge, male, 55 years, facility 3—public)
	*“During our training we are taught to listen and to treat the patient*, *but that did not include advise on their emotions or personal issues”* (facility in-charge, female, 27 years, facility 6—private)
Cultural competency	*“We treat all patients equally no matter what they are financially*, *cultural tribe or religion…all the health workers here have respect for patients and they come from this area*. *We speak to them in Lusoga (local language) and we understand how to treat elders… you notice all of them want to kneel down when they get inside the consultation*, *but if they are elderly*, *I let them know they can just greet me without kneeling”* (health worker, female, 42 years, facility 4—private)

### Role of other actors in the provision of quality primary health care

Each of the six facilities we visited had at least two Village Health Team (VHT) members attached to it. VHT members felt responsible to sensitize the community on health issues, and be an example for the community to follow. To them, the quality of health services provided at the health facilities was rooted in the communication between health workers and patients. Various NGOs were identified as having an impact on the provision of quality health care. These organisations operate at facility, district and national levels, and include organisations such as: Marie Stopes International (provided vouchers for women to access reproductive health services), TASO (support for people living with HIV/AIDS), UNICEF (different projects supporting immunization and child health), WASH (programs improving water and sanitation), etc. Another example is the Uganda National Health Consumers Organisation (UNHCO), responsible for introducing the patients’ rights charter at national level, distributing this information and creating awareness about patients’ rights. Following the establishment of the patients’ rights charter in 2010, the UNHCO has been involved in many projects to empower communities. Specific to improving quality of primary health care are maternal and child health education and advocacy programmes to create awareness of patient rights. In an interview with one of the programme managers, it was said that patients need to be more involved in health policy making and empowered to evaluate the services they receive. They also felt the need to convince health workers and local administrators on this.

Outside the health system, academia is an important group of stakeholders responsible for the training of health care workers in delivering primary health care services. The interviewees in this category were confident that trainees were adequately trained and tested to provide quality clinical care and preventive health services. However, they could not provide data to substantiate this assumption.

### Patient-centered care policies in Uganda

In general, the need to improve quality of primary health care in Uganda was a key driving force behind the development of the policy documents reviewed (see supporting file S1 Table for a list of reviewed documents), with targets set for 2020 to achieve better patient outcomes, health system performance and professional workforce development. However, only one document explicitly mentioned PCC in its title: a donor-driven policy document to improve care of patients with HIV/AIDS [49]. The Health Sector Quality Improvement Framework and Strategic Plan (HSSP, 2015) [33], as well as the Quality Improvement Methods (QIM, 2015) [34] manual both outlined PCC in their objectives. The family medicine curriculums at Makerere and Mbarara universities included a course on community and family-based care where they adopted the eight principles of patient-centered family practice (see supporting file S2 Table).

According to the HSSP and QIM documents mentioned above, patient- and family-centered care appears to be one of the components needed to improve the quality of health care. In these documents, patient- and family-centered care is defined in reference to the Institute of Medicine definition [50]:

“*Health care that establishes a partnership among practitioners*, *patients*, *and their families (when appropriate) to ensure that decisions respect patients wants*, *needs*, *and preferences and that patients have the education and support they require to make decisions and participate in their own care”… It is care that is “respectful of and responsive to individual patient preferences*, *needs*, *and values and ensures that patient values guide all clinical decisions”*. *By involving patients and communities*, *they can contribute to the delivery processes and better manage their health care challenges*.*”* (HSSP–page 9, QIM–page 14)

This definition is then operationalized to include five key components of PCC: “*Dignity and respect*, *choice and empowerment*, *access and support*, *patient involvement in health policy*, *and information sharing”* (QIM–page 11), while patient needs in terms of healthcare are described as follows:

“*Delivered on time by friendly and respectful staff; safe; produce positive result and that they can afford; provide patients with adequate information about their condition and treatment; provide patients with all the medicines they need; give privacy and confidentiality; within patients’ reach (distance) and given in a language they can understand; comfortable; allow continuity of care; provide choice*.*”* (QIM–page 11)

An additional requirement is a health care practitioner who has:

“*Adequate knowledge and skills*, *enough resources (staff*, *medicines*, *supplies*, *equipment*, *transport*, *etc*.*)*, *a good working environment*, *regular training*, *good pay*, *respect / recognition for good work*, *encouragement from colleagues and supervisors*, *access to information*, *feedback and guidance from other levels*.*”* (QIM–page 11)

The documents offer many suggestions on ways to improve the relationship between health workers, patients and the community. However, there are no specified indicators of progress contextualized for a primary health care setting, where most likely one single clinician is attending patients, performing managerial duties, keeping documentation etc. The instances in which outcomes are mentioned are reflective of health system outputs (e.g. reducing waiting time, infection rates, complication rates), and are not linked to patient preferences [51] or experiences (e.g. patient satisfaction or patient enablement–the latter referring to the extent to which a patient is capable of understanding and coping with their health or illness), despite being strongly recommended [26, 40]. Interviews at national level and feedback meetings with the Iganga and Mayuge DHMTs indicated that district level health managers were involved during the writing of both the HSSP and QIM manual for health workers. After publication, copies of both documents were sent to each district followed by a three-day training for district health representatives countrywide. The Iganga and Mayuge DHMTs confirm this in their interviews. The two DHMT’s organized a one-day training towards the end of 2014 to introduce the QIM, and gave a copy to each of the facility-in-charges, which was confirmed by the in-charges. However, no monitoring or trainings have been conducted since then.

### Stakeholder perceptions on patient-centered care

The following data on the perceptions of PCC is presented according to a combination of the Mead and Bower [41] and Stewart et al. [40] models of care distinguishing the following elements of PCC: disease and illness experience, the person-as-a-whole, finding common ground; and the patient-doctor relationship (as illustrated in Fig 1). Table 5 highlights the following sections with associated quotes.

In exploring perceptions of health and illness, most patients indicated that they perceived the health workers being responsible for, and most knowledgeable about, the patients’ health. A few patients also said God was responsible for healing. At home, the household head (in most cases a man) was responsible for making decisions about when and where to seek treatment. The women (mothers, daughters and grandmothers) were responsible for ensuring homes were kept clean, children were fed, and that those who were ill followed the instructions given by the health worker or drug shop owners (the latter being visited when the illness was not perceived as being very severe). Health workers faced challenges in terms of patients’ expression of the cause of their illness, citing patients’ traditional and cultural beliefs. For instance, illness in the Busoga culture is part of everyday life and is even included as part of the morning greeting *(ebikuluma)* which translates to *“how is your sickness”*. These cultural considerations are important in determining the patient preferences and health-seeking behavior (see Table 5).

Understanding the whole person entails looking at the psychological, emotional and social aspects of health care. Patients felt it was not the responsibility of the health worker to discuss emotional and psychological aspects of their health with many saying *“it is disturbing the health worker”*. They appropriated this role to family and friends. Patients did not discuss how their illness was affecting their day-to-day activities at home and were more attentive to instructions on medical treatment as opposed to diet or reducing stress. Health workers reported that there was insufficient time during consultations to discuss psychological or emotional issues with all patients; they usually only discussed emotional issues with patients who were living with HIV, or patients with hypertension who did not adhere to treatment or clearly looked stressed (see Table 5).

Finding common ground (or shared information and decision making) was seen as requiring patients and health workers to have distinct characteristics. Patients’ felt that the communication with the health worker was enhanced when there was trust. This was expressed from pleasant past experiences, kind language, keeping patients’ secrets and providing a private setting for consultation. Patients’ did not feel appreciated when health workers were late and were less likely to express their thoughts about their illness if the health worker was harsh, or did not answer their questions. For instance, patients who felt that the health worker was rude during their first interaction, were less likely to ask what the result of a lab test was. Communication was a recurring theme in interviews with health educators and district health managers, with the assumption that if the health worker could communicate with patients they would have what they need to provide PCC.

On the other hand, health workers viewed *the interaction with patients* as being more complex than just communication. They mentioned several factors that affected the extent to which they involved the patient in decision making. These included the patients’ literacy levels, but also respect and trust between the health worker and the patient, and whether the latter actively asked questions or showed interest in what was being done. The challenge is that while communication is a skill they were taught at medical or nursing school, gaining trust from patients and being able to empathize with their situation are capabilities that are individual to each health worker and require practice. There also seemed to be a strain in communication when there was a difference in the gender or a large age difference between health worker and patient. Health workers also reported losing concentration as the day progressed, especially on days when they had a high number of patents to attend to. With regard to joint decision-making, there were no marked differences in patient interactions between health workers from public or private health facilities; in both settings, it was the health workers who were making most of the diagnostic and treatment decisions.

Health workers were also concerned that patients do not understand the treatment instructions since most of them cannot read. The treatment regime of the drugs were therefore given during the consultation, even though the drugs were normally dispensed by a different person after the consultation. This is contrary to the patients perspectives–while a majority of them did not understand what the results of their lab tests were, they did not ask for an explanation out of fear of being reprimanded for not knowing or not understanding. However, patients did know how to take the medication they had been given. Most patients getting routine care services were familiar with paracetamol tablets, but had little understanding of other types of medication. Patients attending HIV/AIDS clinics in both private and public facilities understood the dosage and purpose of the anti-retroviral or TB drugs that they were taking, and also received advice on diet and sexual health. The adverse effects of medications were never discussed (see Table 5).

The relationship between the health worker and the patient showcases that the health worker holds more power than the patient because of their position and their ability to provide the patient with a diagnosis and treatment. Culturally, women and children also knelt before the health worker as a sign of respect. Health workers had not been separately trained on PCC or elements of it like empathy, mindfulness or being present in the moment. They expressed that their main drive is attending to all the patients that come to the facility in a time-efficient manner. Supervision also plays a key role in guiding the practice of health workers. Health workers complained that most supervision time was spent on clinical indicators and the availability of equipment; despite also having difficulties in staff management or personal issues, the health workers had no one to discuss these issues with or get advice from. They subsequently recommended training on interpersonal skills for health workers and patient engagement together with VHTs, in order to communicate better with their patients and the communities they serve.

Different facility in-charges also mentioned strategies they use to get feedback from the patients on quality of care received at the facility. The most common method is a suggestion box at the exit of the facility. However, in a context where most patients cannot read and write, this is hardly an appropriate approach. Consequently, this only had some success at the semi-urban facilities, with most complaints addressing the rudeness of health workers. In the rural facilities, patients do not utilize suggestion boxes. Both patients and health workers attributed this to challenges in illiteracy, lack of stationery, fear of health worker reactions and the fact that the boxes are located in plain sight, jeopardizing anonymity. Continuity of PCC (outside of patient and health worker interactions) was difficult to explore as it involves the inclusion of families, communities and social welfare organisations that were not included in this study.

## Discussion

The Ministry of Health in Uganda has recognized the need for increased participation of patients and included the provision of Patient-Centered Care as one of its objectives in their Health Sector Quality Improvement Framework & Strategic Plan in 2015 [37]. However, no specific guidelines were outlined to implement PCC or monitor its progress. Consequently, PCC has not fully translated into primary health care practice in Uganda due to individual, community and health system factors that we detail below.

### Developing a patient-centered mindset

Our findings point to a general mutual respect between health workers and patients, albeit with a hierarchical interdependence between them. Several challenges exist that affect the sharing of information between the two sets of actors. Patients felt that health workers are responsible for their health because health workers are perceived to be more educated and have the ability to treat illness. Subsequently, health workers are seen to have more power, both within the health facility environment as well as within the community. During consultations patients only spoke in response to the health workers’ questions or prompting because health workers are perceived to have more knowledge on health matters. In public facilities primarily, negative past experiences and harsh language from rude health workers lead patients to be afraid and withhold information even when it was pertinent to their health and illness. This is in line with other studies conducted in the same field setting that showcase patients making the informed choice to not return to the same facility upon experiencing negative health worker interactions [52].

Patients visiting private facilities reported more confidence in asking questions because they were paying for the services, and felt the health workers are obliged to ‘be nice’ to them and give them what they want. Most patients also feel that it is inappropriate to discuss emotional or psychological issues with their health provider because it is not seen as part of the services offered at a health facility. Additionally, it is also against gendered cultural norms. Patients interact less with health workers who are younger or of the opposite gender, because elders are held in a position of high regard and the younger person is supposed to kneel in respect while greeting their elder. However, this norm is challenged by the health workers’ position of power and we observed old women kneeling to greet younger male health workers. In addition, sexual issues are only discussed among people of the same gender, and therefore patients would keep these issues for another day when a health worker of the same gender would be present. This could also be linked to local cultural perceptions of emotional and psychological illness which are considered more social in nature rather than biomedical, but also to health workers lacking the resources, capacity and/or training to provide adequate care for emotional and psychological issues [53]. A recent review suggests that patients’ expression of pain, identity, and lifestyle can be biased by gender and should be considered as an important part of the patient-provider encounter [54].

Our findings indicate that patients generally do not take responsibility for their own health and are not aware of their rights. Studies in Uganda and elsewhere have shown that patients may be more likely to accept and adhere to treatment when they understand their diagnosis and are involved in their treatment plan [55, 56]. There is also evidence that patient-reported health information interventions and patient education interventions improve healthcare professionals' adherence to recommended clinical practice [56]. Patients are therefore an important resource in improving patient-centeredness, and PCC interventions need to ensure that patients can participate fully by first accepting responsibility for their health.

Health education needs to be accessible even to patients who cannot read visual aids. VHTs can be an additional resource to carry out activities to involve communities in setting their health priorities and enabling patients to understand health information, thereby improving their health literacy. For patients to be able to participate in decision-making, they also need to be aware of their rights. This is a longstanding global challenge [57–60]. Efforts in sub-Saharan Africa have been targeting the publication of patient service and rights charters; in this stride, Uganda too, published its own charter [61]. However, patients interviewed were still not aware of their rights, even when these were posted on facility walls. More innovative and interactive methods that combat illiteracy and complacency are needed to create awareness of patient rights. The lack of understanding of patient rights leads to a perception that patients would not want to understand, or that they are not ‘the experts’. Acknowledging patients’ rights implies a shift to a culture of shared decision-making which will require commitment from patients, health workers and the health system at large [62].

### Integrating interpersonal aspects of care in the training and assessment of health workers

In Uganda, primary health care is mostly provided by nurses and clinical officers. Health workers are trained on the clinical aspects of health care that focus on getting the right diagnosis and prescribing the right medication. They also receive training on community health but this focusses on offering health care to communities through specific single-purposed outreach programmes to promote immunization, reproductive health services, screening for malaria, HIV/AIDS etc. Health workers do receive (some) communication training but admit to having limited skills in demonstrating empathy and gaining patients’ trust. These skills were more developed in health workers who had practiced over longer periods of time, or had experience in working in private facilities where supervision was more strict and mentorship was part of health care practice. Literature indicates that health workers are more likely to fall into traditional hierarchical practice behavior when there is lack of effective supportive supervision and mentorship [63–65], and a high workload [66]. In Uganda, interpersonal aspects of care are not included in assessments that license health workers and no patient dependent assessments exist. Consequently, cases of disrespect, abuse and misconduct, are rarely reported to authorities and health workers are seen to be above the law. Studies warn about the consequences of not having monitoring and evaluation methods in place to collect and analyze patient complaints or even positive feedback [66]. There is a need for continuous training for health workers to develop skills on how to be present in the moment during consultations, actively listen to patients and create an environment where patients feel secure and empowered to explain their preferences and values. This can be incorporated into training and assessments using simulated patients [67, 68]; as well as into practice through mentorship and close supervision of practice [59].

### Strengthening communication and the relationship between patients and health workers

As stated in our findings, health workers are generally respectful and feel that they communicate well with their patients. However, underlying gaps in communication become clearer to pinpoint when the relationship between patients and health workers fares poorly on PCC elements like communication, caring attitudes, empathy, trust and especially shared decision-making. This is exemplified by the fact that despite the majority of patients not understanding the results of their lab tests, they did not ask for an explanation of their results or of the medication they were given, primarily out of fear of being reprimanded by the health worker for not knowing or not understanding.

One of the central factors affecting patient engagement is the challenge it poses to traditional power relations inherent in care relationships and contexts [57]. This could be confronted by encouraging continuous non-dominant, non-hierarchical relationships, through morning health talks at the facility, and dialogue with the community. Health workers need to be trained to create an environment where patients are comfortable to ask questions (by ensuring privacy, prompting for questions and checking whether the patient has understood), and to translate clinical tests or treatment options into a language that patients do understand. Health workers could definitely learn from the training for providers and practice of care for people living with HIV/AIDS. Health workers also reported that they were trained not to get *too* emotionally attached to patients. While compassion fatigue is a legitimate concern when emotional involvement blurs professional conduct [63–65], empathy is considered a necessary requirement to achieve PCC [64]. Epstein et al. [66] further emphasize the need to build the capacities of clinicians to become more self-aware and resilient, and engage in compassionate action.

In general, health workers reported that they are overwhelmed with clinical, administrative and managerial duties; nonetheless, health workers at private facilities showed more polite attitudes towards patients. This is linked to more strict supervision by facility in-charges, administrators and board members; and to the need to be seen as providing good quality health care in order to attract more patients with the ability to pay for services. Incentivized activities, e.g. regular checks, but also additional staff as is the case for people living with HIV/AIDS and patients with diabetes can lead to better relationships between health workers and patients. The latter option, however, is often only available for the ‘privileged’ patients with these specific health problems that capture huge attention from donors and vertical programmes [46, 57–59]. All these challenges are further deepened by a lack of staff assessments and limited supervision capacity at district level with checklists still limited to output data but hardly focusing on processes. Efforts are needed to empower not only front-line health workers, but all actors in the health system, including in-charges, supervisors, district health teams, etc. to take responsibility for the provision and maintenance of good quality primary care.

### Translating policy into practice and encouraging community participation

In Uganda, progress has definitely been made in including PCC as one of the objectives in improving quality of health care [37, 38, 60]. However, this progress hardly trickles down from the national and district policy levels into actual practice. There seems to be a disagreement between donors and Ministry of Health on the model of implementation, leading to a reduction in funding. This, in combination with a lack of patient and health worker involvement in the design of these documents, resulted in poor ownership and uptake of the initiative. The design of the QIM policy documents tend to focus on the health worker as the main agent of change, whereas it is important to also consider the health worker as a person with their own history, specific needs and drivers, and is often working in a challenging environment.

Assessing patients’ perceptions of outcomes is a challenge because most patients reported being satisfied with services received, while simultaneously still complaining not being able to ask questions and not understanding the information in their patient books. This is corroborated by literature indicating the overwhelmingly positive patient satisfaction scores in surveys when questions are framed positively [69], and the need for more comprehensive methods in measuring quality of care [2]. Accordingly, there is a need to employ effective assessment methods that are specific to PCC and include patient-driven outcome assessments of the quality of primary health care. Three (two private and one public) out of the six facilities we visited had a computer to enter patient information which was used to generate monthly reports on utilisation. However, the health information systems could not be used by health workers to access individual patient histories. Patients could also only access the data in their patient books. Health information systems need to be refined so that in addition to compiling reports, they can provide historical information about patients and share data across facilities in a secure way to enable easy access to patient information for effective consultations and referrals.

In line with the existing literature on primary health care interventions in Uganda and other LMICs, stakeholder engagement [57] and particularly community participation [62] in the design and implementation of policies, are important to prevent stagnation of activities in the absence of donor funding. In practice, however, challenges are expected in involving communities in policy-making due to low levels of health literacy, lack of funding for community engagement activities and the lack of methods to show the actual impact of community engagement on policy implementation. Lehmann et al. [70] recommend that the design, implementation and evaluation of community-based interventions by policy makers, whether through the mechanism of VHTs, or not, needs to be located within community health systems which *“include a wide range of actors with*: *relative absence of formal bureaucratic structures and relationships are based on networking and reciprocity; rely on trust and acceptability and are context specific (influenced by local histories*, *economic and political systems*, *and social–cultural norms”* [70].

In summary, PCC is not a clearly shared concept amongst relevant stakeholders in Uganda, but current perceptions highlight that some critical components exist that can be used as stepping stones towards the improvement of quality in health care in this context. These could involve, for instance, empowering patients to know their rights, as well as enhancing information-sharing which would enable them to participate as equal partners in their own health care. In this same regard, health workers could benefit from improved training, that includes aspects of patient-centeredness, accompanied with sustained, supportive supervision. Models of PCC implementation should allow for dynamic and context-specific applications, especially in resource constrained settings where not all components can be implemented concurrently. While PCC is not an ‘all or none’ concept, interventions should ensure the basic pre-requisites: patients are empowered, health workers are competent, and the health system is supportive. Ways forward to ensure the successful implementation of PCC policies into actual practice require for specific indicators to be developed and contextualized to monitor progress in providing patient-centered primary health care.

### Study limitations

Purposive selection of national and district level key informants means that we may have missed out on other relevant players: e.g. actors working at managerial level in the private sector, medical supply companies, social welfare organisations, media, etc. Some of the national level interviewees are insiders to the policy design phase on quality improvement documentation. We tried to counter this by triangulating the information given between different groups of stakeholders and the review of policy documents and newspaper articles. The study of patient-centered care (as compared to people/person centered care) means that patients’ families and members of community were not included in this study. Their perspectives were explored through discussions with VHTs and local council members. Data on patient satisfaction that was collected in patient exit surveys will be presented in another article and these findings can therefore not yet be linked to patient outcomes.

## Conclusions

This study highlights the varying perceptions of patient-centered care amongst Ugandan stakeholder groups. Nonetheless, they agree on the following: the need to involve patients in making decisions about their health, the key role of healthcare workers in that endeavor, and the importance of context in designing and implementing solutions. We conclude with the proposal of three avenues for future action. Firstly, fora that include a wide range of stakeholders may offer a powerful opportunity to gain an inclusive vision on PCC in Uganda. For instance, the stakeholder fora that are advocated for in Ugandan policy documents could be activated, and the issue of PCC could be integrated in their agenda. Secondly, efforts need to be made to ensure that improved communication and information sharing–important components of PCC -translate to actual shared decision making. In that respect, support supervision, monitoring and evaluation of the work of frontline health workers, with due attention for patient-centered care, would constitute a major step forward. Last but not least, the Ugandan health system needs to strengthen its engagement of the transformation from community health workers–VHT’s within the specific Ugandan context–to more comprehensive community health systems. Cross-cutting the entire analysis, is the need to address, in a culturally-sensitive way, the many structural barriers in designing and implementing PCC policies. This is essential in ensuring the sustainable and effective implementation of PCC approaches in low- and middle-income contexts.

## Supporting information

S1 Fig A representation of the health care system of Uganda showing the administrative, service delivery and regulatory arrangements. A figure presenting the levels of health care in the Ugandan health system and how they interact with each other according to the Primary health care systems (PRIMASYS): case study from Uganda [71].
(DOCX)

S2 Fig Media articles on the nationwide nurses’ strikes in Uganda during the period of data collection. (DOCX)

S1 Table List of policy documents included in content analysis. A list of documents from the Uganda Ministry of Health and other organisations that are involved in the provision of primary health care in Uganda, and mention patient centered care.
(DOCX)

S2 Table Principles of patient-centered care as seen in the family medicine curriculum. (DOCX)

S1 Appendix Samples of questions asked and tools used during in-depth interviews and focus group discussions with stakeholders. A compilation of the informed consent forms and tools used in qualitative and quantitative data collection (tools used for collecting data from patients were translated into Lusoga).
(DOCX)

## Abbreviations

CHW: Community Health Worker
DHMT: District Health Management Team
FGD: Focus Group Discussion
HC: Health Centre
HIV/AIDS: Human Immunodeficiency Virus infection and Acquired Immune Deficiency Syndrome
HSSP: Health Sector Quality Improvement Framework and Strategic Plan
IDI: In-Depth Interview
IMHDSS: Iganga Mayuge Health and Demographic Surveillance Site
LMIC: Low and Middle Income Country
MCH: Maternal and Child Health
NCD: Non-Communicable Disease
NGO: Non-Governmental Organization
NVivo: Qualitative data analysis computer software package produced by QSR International
PCC: Patient-Centered Care
PI: Principal Investigator
QIM: Quality Improvement Methods: a manual for health workers in Uganda
TASO: The AIDS Support Organisation of Uganda
TB: Tuberculosis
UNHCO: Uganda National Health Consumers Organisation
UNICEF: United Nations International Children's Emergency Fund
VHT: Village Health Team
WASH: Water, Sanitation and Hygiene
