# Supplementary material for: Stakeholder perceptions on patient-centered care at primary health care level in rural eastern Uganda: A qualitative inquiry
Source: PLoS One. 2019 Aug 28;14(8):e0221649. doi: 10.1371/journal.pone.0221649 (PMC6713356; doi:10.1371/journal.pone.0221649)
Supplement: S2 Fig — (DOCX) [file pone.0221649.s002.docx]

S2 Fig. Media articles on the nationwide nurses’ strikes in Uganda during the period of data collection **
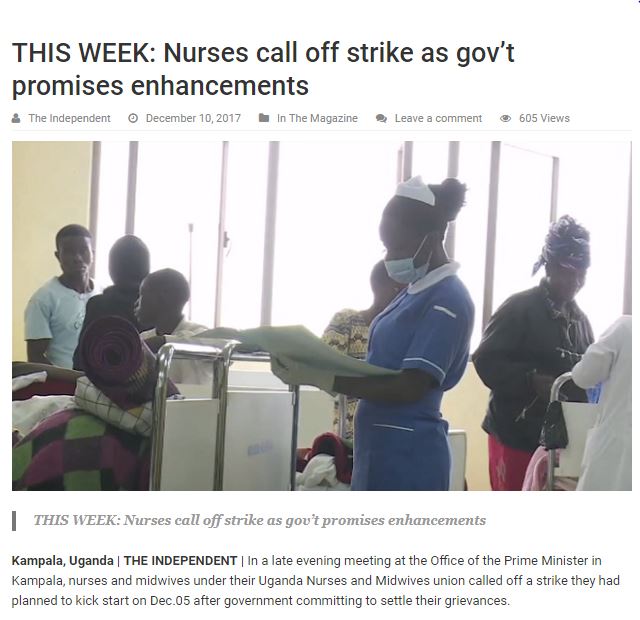
**

*Source the Independent observer*

<https://observer.ug/news/headlines/56297-nurses-call-off-planned-strike-after-meeting-museveni.html>

**
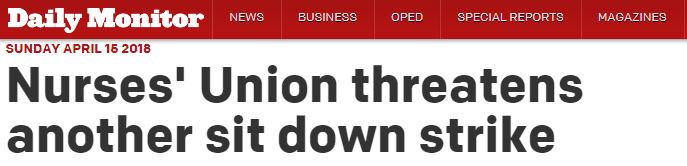
**

**
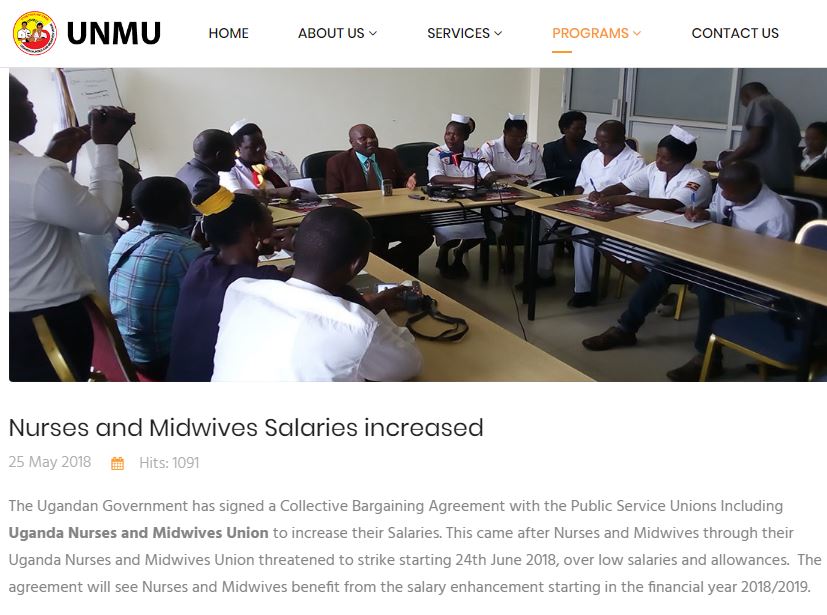
***Source: the Uganda Nurses and Midwives Union website* [**http://www.unmu.ug/index.php/blog/unmu-calender-events-news/17-nurses-and-midwives-salaries-increased**](http://www.unmu.ug/index.php/blog/unmu-calender-events-news/17-nurses-and-midwives-salaries-increased)

**Other links to videos show the effects of the health worker strikes at Iganga hospital and the end of the nurses strike respectively.**

[**https://www.youtube.com/watch?v=ivI_uUS13AM**](https://www.youtube.com/watch?v=ivI_uUS13AM)

[**https://www.youtube.com/watch?v=lnfvww3O5VM**](https://www.youtube.com/watch?v=lnfvww3O5VM)
