# Supplementary material for: Stakeholder perceptions on patient-centered care at primary health care level in rural eastern Uganda: A qualitative inquiry
Source: PLoS One. 2019 Aug 28;14(8):e0221649. doi: 10.1371/journal.pone.0221649 (PMC6713356; doi:10.1371/journal.pone.0221649)
Supplement: S1 Table — A list of documents from the Uganda Ministry of Health and other organisations that are involved in the provision of primary health care in Uganda, and mention patient centered care. (DOCX) [file pone.0221649.s003.docx]

## S1 Table. List of policy documents included in content analysis. A list of documents from the Uganda Ministry of Health and other organisations that are involved in the provision of primary health care in Uganda, and mention patient centered care.

| **Document source organisation** | **Title** |
| --- | --- |
| Ministry of Health (MoH), USAIDS Assist programme | Health sector quality improvement framework and strategic plan (2015/16 – 2019/20)  (HSSP) |
| MoH, USAIDS Assist programme | The quality improvement methods: a manual for health workers in Uganda (QIM) |
| MoH | Uganda Health Sector Development Plan (2015/16 – 2019/20)  (HSDP) |
| MoH | Strategy For Improving Health Service Delivery 2016-2021: presidential directives for health sector/ service improvements to attain middle income status by 2020 (HSDD) |
| MoH | Uganda clinical guidelines: National Guidelines for Management of Common Conditions (CG) |
| MoH: Quality assurance department, UNHCO | Patients’ Charter (PC) |
| Makerere University | Family Medicine Training curriculum (FM) |
| Uganda AIDS Commission, MoH | National HIV/AIDS Strategic Plan, Monitoring And Evaluation Plan And Indicators’ Handbook (2015/2016 - 2019/2020) |
| USAIDS, PEPFAR | A Comprehensive and Patient-centered Approach to Improving Chronic HIV Care and Treatment in Uganda : Health Care Improvement Project |
| MoH | The National Integrated Early Childhood Development Policy Action Plan Of Uganda (2016-2021) |
| MoH | Insights from a National Health Care Quality Improvement Strategy Meeting |
