# Supplementary material for: Stakeholder perceptions on patient-centered care at primary health care level in rural eastern Uganda: A qualitative inquiry
Source: PLoS One. 2019 Aug 28;14(8):e0221649. doi: 10.1371/journal.pone.0221649 (PMC6713356; doi:10.1371/journal.pone.0221649)
Supplement: S2 Table — (DOCX) [file pone.0221649.s004.docx]

## S2 Table. Principles of patient-centered care as seen in the family medicine curriculum

| The Institute of Medicine defines patient-centered care as: “Providing care that is respectful of, and responsive to, individual patient preferences, needs and values, and ensuring that patient values guide all clinical decisions [12]. | |
| --- | --- |
|  | **Principles of patient-centered care** |
| **1.** | **Respect for patients’ values, preferences and expressed needs by: i**nvolving patients in decision-making, recognizing they are individuals with their own unique values and preferences. Treat patients with dignity, respect and sensitivity to his/her cultural values and autonomy. |
| **2.** | **Coordination and integration of care including:** clinical care, front-line patient care, ancillary and support services. |
| **3.** | **Information and education by s**haring information between health workers and patients on: clinical status, progress and prognosis; processes of care; information to facilitate autonomy, self-care and health promotion. |
| **4.** | **Physical comfort including:** pain management, assistance/advice with activities and daily living needs; influence of hospital surroundings and environment**.** |
| **5.** | **Emotional support and alleviation of fear and anxiety over:** physical status, treatment and prognosis; the impact of the illness on themselves and family; and the financial impact of illness. |
| **6.** | **Involvement of family and friends:** addressing the role of family and friends in the patient experience by: providing spaces for family and friends, involving family and close friends in decision making, supporting family members as caregivers and recognizing the needs of family and friends. |
| **7.** | **Continuity and transition:** patients ability to care for themselves after discharge. |
| **8.** | **Access to care:** distance to facility, transport, scheduling and availability of appointments, referrals and speciality services. |

*Source: Community health curriculum (adopted from eight principles of patient-centered care highlighted in research conducted by the Picker Institute and Harvard Medical School).*
