# Supplementary material for: Stakeholder perceptions on patient-centered care at primary health care level in rural eastern Uganda: A qualitative inquiry
Source: PLoS One. 2019 Aug 28;14(8):e0221649. doi: 10.1371/journal.pone.0221649 (PMC6713356; doi:10.1371/journal.pone.0221649)
Supplement: S1 Appendix — A compilation of the informed consent forms and tools used in qualitative and quantitative data collection (tools used for collecting data from patients were translated into Lusoga). (DOCX) [file pone.0221649.s005.docx]

## S1 Appendix. Samples of questions asked and tools used during in-depth interviews and focus group discussions with stakeholders. A representation of the various topics that were covered and tools used during in-depth interviews with patients, health practitioners, health managers, health educators and health policy makers.

**SAMPLE QUESTIONS FOR INTERVIEWS WITH HEALTH PRACTITIONERS, HEALTH MANAGERS, HEALTH EDUCATORS AND HEALTH POLICY MAKERS (CONDUCTED IN ENGLISH)**

| **Research topic** | **Questions asked to health managers, health educators and health policy makers** |
| --- | --- |
| Perceived roles of stakeholders | What do you feel is your role in the provision of primary health care?   - What enables you in carrying our your roles? - What challenges you in carrying out your roles |
| Perception of quality of care | What do you think about the current quality of primary health care services in Uganda?   - And what about: at the facility you visited? (for patients), in your facility? (for in-charges), at your district? (for district health managers) |
| Conceptualisation of PCC | Have you ever heard of the term patient-centered care?  What do you think it means?  **Operational definition read out was:**  Patient-centered care is an approach to providing health care with the following key characteristics:   1. Explores the patients reason for a visit, concerns and need for information (their perception of health or illness) 2. Seeks an understanding of the patient as a whole person, their physical, psychological, emotional and social needs (multiple dimensions of illness) 3. Finds common ground between the health worker and the patient for diagnosis and management of illness 4. Enhances prevention and health promotion 5. Promotes a continuous relationship and shared decision making between health workers and patients 6. Explores health worker perceptions of their role in health care (importance of creating trust and empathy); and what has influenced their perceptions and practice (training, experience, culture, supervision)   Which of these aspects do you think is important in the provision of good quality primary health care and why? |

**Tool: INFORMATION SHEET FOR INTERVIEWS WITH HEALTH MANAGERS, HEALTH EDUCATORS, PRIMARY LEVEL HEALTH CARE WORKERS AND PATIENT ORGANISATION REPRESENTATIVES**

**Understanding patient-centered care approaches at primary health care level in sub-Saharan Africa: the case of Iganga district in Uganda**

My name is Evelyn Waweru a research student at the Institute of Tropical Medicine in Antwerp. The ITM is one of the training and research institutions that works with many scientific institutions, governments and organisations all over the world for long-lasting improvement of health care and disease control in developing countries.

You are invited to take part in a research study looking at approaches that can be taken to ensure the care provided at primary health care facilities is more focussed on the patient; and what challenges and opportunities would be experienced*.* Before you decide to participate in this study, it is important that you read this form. The available alternatives and the right to withdraw your consent to participate at any time are described below. You have the right to ask questions at any time.

**Purpose and description of the study**

This is a social-scientific study to explore current initiatives, opportunities and challenges in the delivery of patient centered primary health care services in Uganda. The data will contribute to added knowledge on tools to understand the factors that affect the quality of primary health care and the feasibility of patient-centred care approaches at primary health care level in sub-Saharan Africa.

The planned study in Uganda will begin with a baseline situational analysis, where data will be collected at various levels and across 6 facilities; with health workers, health educators and 180 patients over a period of one year. If you accept to participate in this study, you will be shadowed (observed) and later asked questions on how you feel about the interaction between you and your patients/students; and your ability to make joint decisions to maintain their health or manage their illness.

**Sponsor of the study**

The sponsor of the study is the Trans Global Health Erasmus Mundus Joint PhD Programme, working together with universities in Europe (ITM; VU) and in Uganda (Makerere) up to 2019.

**Voluntary participation**

You participate entirely voluntarily in this study and you have the right to refuse to participate in the study. Your decision to participate in this study or not, will have no influence whatsoever on your work at this facility. You also have the right to stop you participation in the study at any time, even after you have signed the consent form. You do not have to give a reason for withdrawing your consent to participate. The withdrawal of your consent will not cause any disadvantage of loss of advantages. If you accept to participate in this study, you will receive this information form to keep it and you will be asked to sign the attached consent form.

**Risks and inconveniences**

There will be no physical risks to participating in this study. All data will be coded such that the identity of the participant is not revealed but feedback is possible. You also have the right to access the information you provide to us and to make any adjustments if necessary. They may be social or psychological risks in the time taken for the interview or sensitive information discussed during the interview but confidentiality will be maintained by the research team.

**Advantages**

We cannot confirm that you will personally benefit directly from your participation in this study. If you consent to participate in this study, the information resulting from this study can contribute to better knowledge on the interaction between you and your patients and help other patients in future.

**Compensation**

There is no monetary compensation available for this study.

**Protection of your private life**

Your identity and your participation to this study will be treated strictly confidential. You will not be identified by name or in any other identifying manner in files, results or publication concerning this study. Your identity remains secret since personal information will only be designated by a unique participant number (therefore coded).

**Ethics committee**

This study has been reviewed by the Institute of tropical medicine PhD committee, Makerere University Institutional Review Board and The Uganda National Council For Science And Technology. If the data collected in this study will be used for another study, the latter will be submitted again to all the relevant ethical committees for review.

**Contact persons in the case of questions concerning the study**

If you think having incurred damage related to the study or if you have questions concerning the study or your rights as a participant, you can contact, now, during or after the study:

Study Investigator: Everlyn Waweru Telephone: +256 771 905168 or +32 486 74 96 95

Email: [ewaweru@itg.be](mailto:ewaweru@itg.be)

Study Supervisor: Prof. Bart Criel

Institute of Tropical Medicine; Unit of Equity and Health

Email: [bcriel@itg.be](mailto:bcriel@itg.be)

IRB chairperson: Dr. Suzanne Kiwanuka,

skiwanuka@musph.ac.ug, +256-701-888-163/ 256-312-291-397

**TOOL: CONSENT FORM FOR KEY INFORMANT INTERVIEWS WITH HEALTH MANGERS, HEALTH EDUCATORS, PRIMARY LEVEL HEALTH CARE WORKERS AND PATIENT ORGANISATION REPRESENTATIVES**

| **Understanding patient-centered care approaches at primary health care level in sub-Saharan Africa: the case of Iganga district in Uganda**  **Lay explanation:** A study looking at approaches that can be taken to ensure the care provided at primary health care facilities is more focussed on the patient; and explore what challenges and opportunities would be experienced |
| --- |

| ***Part which is to be reviewed and signed by the interviewee (health worker / district management officer/health organisation representative)***  I, (undersigned, name and first name) __________________ confirm that I have been informed about the study and that I have received a copy of the information sheet and the consent form. I have read and understood the information. The researcher has given me sufficient information concerning the conditions and the length of the study. In addition, I have received sufficient time to consider the information and to ask questions, to which I have received satisfying answers.  – I have understood that I can put to a stop my participation in this study at any time after having informed the researcher about this, and that this decision will not cause any disadvantage.  – I agree with the collection, the processing and the use of these observational data, as described in the patient information sheet. I also agree with the transfer and the processing of these data in other countries than Belgium.   - I agree with the use by the promotor of these coded data for other research purposes. - I consent voluntarily to participate in this study and to cooperate in all the examination requested. I am willing to give information concerning my practice, and interaction with patients.   – I agree that my patients and healthcare professionals involved in providing health care at this facility are informed about my participation in this study and as such:  1) Give consent / do not give consent* to take part in the study  2) Give consent/ do not give consent* to be observed in my practice / consultations  3) Give consent/ do not give consent* to having the interview tape recorded  3) Give consent / do not give consent* to be anonymously quoted  (* Delete as appropriate).  I understand that I can change my mind at any stage and it will not affect me in any way.  ***Date: ___________ Signature participant: _____________*** | |
| --- | --- |
| Part only destined to the investigator I, undersigned, __________________________ confirm that I have informed, ________________ (full name of the participant) and that he/she has consented to participate in the study. Date: _____________ Signature: ___________________ |  |

**Tool 2e: INTERVIEW GUIDE FOR IN-DEPTH INTERVIEWS WITH HEALTH MANGERS, HEALTH EDUCATORS, PRIMARY LEVEL HEALTH CARE WORKERS AND PATIENT ORGANISATION REPRESENTATIVES**

**Understanding patient-centered care approaches at primary health care level in sub-Saharan Africa: the case of Iganga district in Uganda**

**INTRODUCTION**

- The need to improve the quality of care offered at primary health care level has been an important part of health sector reforms in Uganda promoting equity in health and better functioning of health systems.
- More recently, there has been a move to further involve communities in the decision-making process for health care services
- Literature shows important benefits of providing care that is tailored to patient needs and preferences: improved communication can improve satisfaction and biomedical outcomes
- This study aims to identify the relevance of patient centered care as a strategy to improve the provision of health care at the primary health care level by emphasising a collaborative and respectful partnership between health providers and consumers

1. **Key informant profile**

What is your role in the provision of primary health care?

- Probe on specific roles, training, qualifications and services they provide
- Probe further if involved in provision of patient centred care

1. **Exploring perceptions about the services provided at primary health care facilities**

What is your understanding of the services offered in this facility?

- What is the list of services?
- Who are the key players at district, facility and community level?
- What is your perception of other types of primary health facilities? (public/private, profit/not-for-profit, family practice)

1. **Exploring perceptions about the quality of health care provided at primary health care (PHC) facilities in general**

What is your perception of the quality of care offered at primary health facilities?

- What is the state of resources for primary health care facilities? e.g. medicine, equipment, non-pharmaceutical supplies, consultation rooms / space
- Perception of the characteristics of staff working in PHC facilities: training, qualifications, experiencecompensation, any incentives to work in rural areas, staff turn over
- What is considered good practice in a clinical consultation? is this reflected in clinical practice guidelines?
- How is the quality of care evaluated? e.g. supervision by the district level
- What other factors determine the quality of care offered? waiting time, opening hours, access to the catchment population, associated fees, e.t.c
- Is there any difference in the quality of care offered in public, private health care facilities? what about traditional health providers?

1. **Exploring perceptions about the quality of health care provided at particular primary health care (PHC) facility visited and specific to patient centredness (Stewart et;al)**

- **For this facility (in case not mentioned in the above question)**
  - What is the state of resources
  - What staff work here
  - Are there any guidelies for practice?
  - Do you have supervisory support?
- In your daily practice,
  - How accessible is the facility and health workers to the patients
    - opening hours
    - waiting times
  - How do you ensure good practice in your clinical method: diagnosis, physical examination and treatment
  - Do you think aspects of your training or experience or background affect your interaction with patients? and how? motivation?
  - Is health education and promotion a part of your practice?
  - What about communication: asking and answering questions, establishing trust respect and empathy?
  - Do you think its important to have knowledge of the multi-dimensional aspects of the patient and their family? how do you get this information during consultations?
  - to what extent is the capacity of patients or caregivers, as well as the burden of treatment on the patient-caregiver unit, kept in mind?
  - What is the capacity of caregiver/ family/ environment/ overall situation to fulfil caregiving tasks/provide adequate support/care to the patient?
  - How is this related to self-efficacy?
- What challenges have you faced in providing quality care at this facility?
  - Do you feel understood as a person?
  - Probe on resources available, effective use of time, supervision
  - Role of medical records
- What opportunities exist to improve the quality of care in primary health care facilities?
  - probe on awareness of patients’ perceptions of health care services, consultations and preferences?

1. **Are there any other important activities or events that have happened or are happening that have an impact on the quality of care offered in this primary health care facility?**

**Probe on:**

- NGOs
- Patient organisations
- Community resource persons linked to the facility e.g. community health volunteers

1. **I would like to introduce you to a concept called patient centered care.**

Our concept of patient centered care

An approach to providing health care with the following key characteristics:

1. Explores the patients reason for a visit, concerns and need for information(and their perception of health or illness)
2. Seeks an understanding of the patient as a whole person, their physical, emotional and social needs - multiple dimensions of illness;
3. Finds common ground for diagnosis and management of illness
4. Enhances prevention and health promotion
5. Promotes a continuous relationship and shared decision making between health practitioners and patients

**Do you think this is an important concept in the provision of primary health care? and why?**

**If so:**

- What key areas should a PCC intervention focus on?
- What resources would be needed?
- Who would/should be involved?
- How it be evaluated?

**Additional comments to check**

*What would you be interested in learning through our work?*

*Any specific questions/areas to cover to inform PCC implementation in a transitioning health system?*

*Anyone else we should talk to?Contacts of community health volunteers or patient organisations or s linked to the facilities*

**Conclusion**: Thank the interviewee for their participation and conclude the interview.

**TOOLS FOR PATIENTS (TRANSLATED IN LUSOGA)**

**PATIENT INTRODUCTION SHEET** *(in the waiting room before entry to the consultation room, this can also be done by the health worker before the consultation)*

*Research title***:**

Okutegeera engeri edh’endhidandaba eyetololera ku mulwaire ku mutendera gw’amalwaliro agasookerwaku mu maserengeta g’eirungu lya Sahara-Ensonga ya Uganda

*Understanding patient-centred care approaches at the level of primary health care facilities in sub-Saharan Africa: the case of Uganda*

**Enhandula**

**Bwekiba kikolebwa alikunoonereza**

Okusookera irala, webale inho olw’ebiserabyo niinze wano buti. Amainha gange nhinze__________________anoonereza okuva mu Institute of Tropical Medicine mu Antwerp. Mu Uganda tuli kukolera ghalala ni Makerere University. Tuli kukola okunoonereza ku ngeri edhisobola okukozesebwa okukakasa nga empeereza eweebwa mu malwaliro agasookerwaku eta inho eisira ku mulwaire; era buzibu na mikisaki byetunasobola okubitamu. Nandienze okubuzaaku ebibuuzo ebyekuusa ku kino nga omaze okwebuzaaku, kino bwekiba kirungi ndidha kukulindira ku___________________(kuluya aghasembayo era nkukulemberemu mu kifo eky’ekusifu muno) nga omaze okuyaalira omusawo, tubonagane.

*Introduction*

*If done by the researcher*

*First of all, thank you very much for your time with me right now .My name is _______________a research assistant working for the Institute of Tropical Medicine in Antwerp. In Uganda we are working together with Makerere University*

*We are conducting a research study looking at approaches that can be taken to ensure the care provided at primary health care facilities is more focussed on the patient; and what challenges and opportunities would be experienced. I would like to ask you some questions in regard to this after your consultation, if this is okay with you, I will be waiting for you at _________________(outside the last point of care and guided to a private location within the facility) after your visit with the health worker, see you soon* ***.***

Bwekiba kikolebwa omusawo (okugeza nga bamaze okwogera kuby’obulamu)

Tusangaire okubonaku olwaleero ku irwaliro, bano n’abanoonereza nga balikolera aba Insititute of Tropical Medicine mu Antwerp ne eitendekero eikulu ery’e Makerere.

*If done by the health worker (for example after a health talk)*

*Welcome to the facility today, these are research assistants working for the Institute of Tropical Medicine in Antwerp and Makerere University*

Bali kukola okunoonereza ku ngeri edhisobola okukozesebwa okukakasa nga empeereza eweebwa mu malwaliro agasookerwaku eta inho eisira ku mulwaire; era buzibu na mikisaki byetunasobola okubitamu. Bandienze okubuuzaaku ebibuuzo ebyekuusa ku kino nga omaze okwebuzaaku, kino bwekiba kirungi baidha kukulindira ku___________________(kuluya webaidhandabira awasembayo era baidha kukulagirira era bakulemberemu mu kifo eky’ekusifu muno) nga omaze okukyaalira omusawo, webale olw’enkolaganayo.

*They are conducting a research study looking at approaches that can be taken to ensure the care provided at primary health care facilities is more focussed on the patient; and what challenges and opportunities would be experienced. They would like to ask you some questions in regard to this after your consultation, if this is okay with you, they will be waiting for you at _________________(outside the last point of care and guided to a private location within the facility) after your visit with the health worker, thank you for your cooperation.*

**Patient Information Sheet (after receiving care)**

Okutegeera endabirira ey’abalwaire ku mutendera ogw’amalwaliro agasookerwaku mu maserengeta ga Africa: Ensonga ya Uganda

*Understanding patient-centred care approaches at the level of primary health care facilities in sub-Saharan Africa: the case of Uganda*

**Enhinonola enhangu ku kunonenereza**

Kuno n’okunoonereza okuli kulingirira ku ngeri edhisobola okukozesebwa okukakasa nga empeereza eweebwa mu malwaliro agasookerwaku eringa inho ku mulwaire; era buzibu na mikisaki byetunasobola okubitamu ku malwaliro agasookerwaku mu maserengeta ga Sahara

*Lay explanation of research title:*

*This is a study looking at approaches that can be taken to ensure the care provided at primary health care facilities is more focussed on the patient; and what challenges and opportunities would be experienced at primary health care level in a Sub-Saharan context*

Okusookera irala, webale inho olw’ebiserabyo okutyama niinze wano buti. Amainha gange nhinze__________________anoonereza okuva mu itendekero lya Makerere University

*First of all, thank you very much for your time in sitting down with me right now .My name is _______________a research assistant working with Makerere University*

Tuli kukola okunoonereza ku ngeri edhisobola okukozesebwa okukakasa nga empeereza eweebwa mu malwaliro agasookerwaku eringa inho ku mulwaire; era buzibu na mikisaki byetunasobola okubitamu. Nandienze okubuuzaaku ebibuuzo ebyekuusa ku kino.Nenda otegeere nti ghazira kituufu oba kifu mu buli ky’okoba. Era, okunoonereza kuno tikuja kumanibwa. Kino kitegeeza nti buli ky’okoba tikiidha kukunoonenkerezebwaaku. Okunoonereza kwidha kutwala kitundu kya saawa. Kinaaba kirungi singa mpandiika byonandiramu? Nga bwobona, nnina olupapula n’ekalamu okuwandiika, kino kidha kunnamba okwiidhukira. Kansubire nga tofaayo.

*We are conducting a research study looking at approaches that can be taken to ensure the care provided at primary health care facilities is more focussed on the patient; and what challenges and opportunities would be experienced. I would like to ask you some questions in regard to this. I would just like you to know that there is no right or wrong in anything you say. Also, this survey is anonymous. This means that anything you say will not be traceable back to you. The survey will take about a half an hour. Would it be okay if I wrote your answers down? As you can see, I have some paper and pencil to write, this will help me to remember. I hope you do not mind.*

Nga okaali kusalagho kwenhigira mu kunoonereza kuno, kyamugaso okusoma olupapula luno. Olina eidembe okubuuza ebibuuzo ekiseera kyonakyona. Wandienze mbitte mu lupapula luno niighe? *[if yes, Continue with this informed consent form, if no terminate the conversation here and record refusal]*

*Before you decide to participate in this study, it is important that you read this form. You have the right to ask questions at any time. Would you like me to go through the form with you? [if yes, Continue with this informed consent form, if no terminate the conversation here and record refusal]*

**Omugaso n’okwinhonola ku kunoonereza**

Kuno n’okunoonereza okuzuula emikisa n’obuzibu mu kutuusa endabirira ey’abalwaire mu malwaliro agasookerwaku mu Uganda. Twidha kuba nga twogera n’abalwaire, abakola mu by’obulamu (abasawo n’abakola egy’okuwereza abantu), abakola amateeka, abasomesa eby’obulamu era n’abanoonereza abenhigira mu malwaliro munaana mu Uganda. N’olwekyo, buli gwekigemaku aidha kwetebwa okwenhigira mu kutegeka n’okukola engeri edh’okutaasa edhigwana, emitendera egy’okulondoola gyidha kukolebwa okupima enkyuuka etereibwabwo okutaasa ku mutindo ogw’empeereza eweebwa mu bifo ebidhandabirwamu ebya ga gavumenti n’eby’obwanakyeegha.

*Purpose and description of the study*

*This is a study to explore opportunities and challenges in the delivery of patient-centered primary health care services in Uganda. We shall be talking with patients, health care workers (doctors, nurses and social workers), policy makers, health educators and researchers involved in primary level care at 8 facilities in Uganda. Consequently, all stakeholders will be invited to participate in the design and implementation of an appropriate intervention, follow-up phases will be conducted to assess the impact of interventions on the quality of versatile health care services provided at both public and private primary care facilities*

Bwoikiriza okwenhigira mukunoonereza kuno, oidha kubuuzibwa ebibuuzo kungeri gyowuliramu kukutabagana kwo n’omusawo wo, era n’obusobozi bwo okukola okusalawo okwaghalala okukuuma obulamu bwo oba okusobola obulwairebwo.

*If you accept to participate in this study, you will be asked questions on how you feel about the interaction between you and your health care provider; and your ability to make joint decisions to maintain your health or manage your illness*.

**Okwenhigiramu okwekyeyendeire**

Wenhigiramu kyeyendeire mu kunoonereza kuno era olina eidembe okudhema okwenhigira mu kunoonereza kuno. Okusalawokwo okwenhigira mu kunoonereza kuno oba bbe, tikwiidha kuba na kyamaanhi kyekukola ku bwidhandabi bw’oduna ku irwaliro. Era olina eidembe okulekera okwenhigira mu kunoonereza kuno ku kiseera kyonakyona, nibwooba oikiriza.

***Voluntary participation***

*You participate entirely voluntarily in this study and you have the right to refuse to participate in the study. Your decision to participate in this study or not, will have no influence whatsoever on the care you get at this facility. You also have the right to stop you participation in the study at any time, even after you have given consent.*

**Obuzibu n’obukaluubirivu**

Tighaabe bukaluubirivu okwenhigira mu kunoonereza kuno.

*Risks and inconveniences*

*There will be no physical risks to participating in this study. All data will be coded such that the identity of the participant is not revealed but feedback is possible. You also have the right to access the information you provide to us and to make any adjustments if necessary. They may be social or psychological risks in the time taken for the interview or sensitive information discussed during the interview but confidentiality will be maintained by the research team.*

**Ebirungi**

Titusobola kukakasa nti oidha kuganulwa mu buligho okuva mu kwenhigira mu kunoonereza kuno leero. Bwoikiriza okwenhigira mu kunoonereza kuno, amawulire okuva mu kunoonereza kuno gasobola okuyamba mu kulongosa amagezi ku kwogerezagania wagatiwo n’abalwairebo era n’okuyamba abalwaire abandi yebwiidha.

*Advantages*

*We cannot confirm that you will personally benefit directly from your participation in this study today. If you consent to participate in this study, the information resulting from this study can contribute to better knowledge on the interaction between you and your patients and help other patients in future****.***

**Okuliyirwa**

Ghazira kuliyirwa mu sente kuliwo kulw’okunoonereza kuno leero kubanganokubuuzibwa kuli kukolebwa ku irwaliro, aye osaana wenhigire mu kunoonereza okwiiraku okwaghalala; oidha kusasulwa entambula.

***Compensation***

***There is no monetary compensation available for this study today because the interview is done at the facility, but should you agree to participate in the subsequent focus group discussion; you will receive a transport reimbursement***

**Okukuuma obulamubwo obw’ekyaama**

Ebikugemaku n’okwenhigirakwo mu kunoonereza kuno biidha kutwalibwa nga bya kyaama. Toidha kumanhikibwa ku maina oba mu ngeri eyindi ey’okumanibwa mu mpapula, ebinaava oba ebinafulumizibwa nga bigema ku kunoonereza kuno. Ebikugemaku bisigala nga kyaama olw’okubanga amawulire ku muntu agidha kutebwaaku namba eyendhawulo (n’olwekyo gaidha kukwekwebwa). Amawulire agakugemaku gaidha kusengedhebwa era gekeeneeneezibwe n’ebyuuma ( mu kyuuma ki kalimagezi) oba mu ngeri ya buliidho okusobola okusalawo ebinaava mu kunoonereza kuno. Era olina eidembe okusaba anoonereza okubona ku mawulirego era n’okugatereeza bwekiba kyetagisa. Okukuuma amawulire ag’omuntu kitebwaawo mu iteeka ery’omwezi gwa December nga 8 omwaka 1992 erigema ku kuuma ekyaama

*Protection of your private life*

*Your identity and your participation to this study will be treated strictly confidential. You will not be identified by name or in any other identifying manner in files, results or publication concerning this study. Your identity remains secret since personal information will only be designated by a unique participant number (therefore coded).*

**Akakiiko ak’empisa**

Okunoonereza kuno kwetegerezeibwa akakiiko k’abakenkufu ku k’eitendekero lya tropical medicine

*Ethics committee*

*This study has been reviewed by the Institute of tropical medicine PhD committee* *Makerere University Institutional Review Board and The Uganda National Council For Science And Technology.*

**Abantu ab’ebuuzibwaku singa wabagho ebibuuzoebigema ku kunoonereza**

Bw’olowooza nga ofuniemu okukosebwa okwekuusa ku kunoonereza oba bwoba n’ebibuuzo ebigema ku kunoonereza oba eidembelyo nga eyenhigiremu, osobola okutuukirira, buti, mu kiseera eky’okunoonereza oba nga okunoonereza kuwoire.

***Contact persons in the case of questions concerning the study***

*If you think you have incur damage related to the study or if you have questions concerning the study or your rights as a participant, you can contact, now, during or after the study:*

Study Investigator: EverlynWaweru Telephone: +32 486 74 96 95 or +256 771 905168

Email: [ewaweru@itg.be](mailto:ewaweru@itg.be)

Study Supervisor: Prof. Bart Criel; Institute of Tropical Medicine; Unit of Equity and Health

Email: [bcriel@itg.be](mailto:bcriel@itg.be)

IRB chairperson: Dr. Suzanne Kiwanuka,

skiwanuka@musph.ac.ug, 256-701-888-163/ 256-312-291-397

**PATIENT EXIT INTERVIEW QUESTIONNAIRE (Translated to Lusoga)**

Okutegeera engeri edh’endhidandaba eyetololera ku mulwaire ku mutendera gw’amalwaliro agasokerwaku mu maserengeta ga eirungu lya Sahara-Ensonga ya Uganda

*Understanding patient-centred care approaches at the level of primary health care facilities in sub-Saharan Africa: the case of Uganda*

**(Interviewer to fill in this information before proceeding with interview)**

| 1.0 | Date of interview | DAY [__][__] MONTH [__][__]YEAR[__][__][__][__] |
| --- | --- | --- |
| 1.1 | Interviewer code / Initials | [__][__] |
| 1.2 | Name of facility | ___________________________________ |
| 1.3 | District name |  |

| 1.4 | Waligho omulwaire gw’osobola okubuuza?  *Is there a potential patient to interview?* | YES …………………………………… 1  NO ……………………………………. 2 |  |
| --- | --- | --- | --- |

**Inclusion Questions: Circle as**

| - Bakukozeeku?   ***Have you been attended to?*** | Y/N |
| --- | --- |
| - Otera okuviira mu kitundu kino?   ***Do you normally reside in this area?*** | Y/N |
| - Obaire oviira mu kitundu kino okumala emyezi omukaaga egyibisegho?   ***Have you lived in this area for the last six months?*** | Y/N |
|  |  |
| - Ali kubuzibwa aweza emyaka eikumi n’omunaana n’okuswiika?   ***Is the interviewee 18 years and over?***  ***Mubuuze emyaakagye bwoba tiwekakasa***.  Ask their age if you are not sure.  ***Bwaba taweza myaka ikumi na mukaaga (16), tobuuza.***  **If below 16 years of age, do not interview** | Y/N |

[AKAGHAYIRO AKAMUTOLAMU] BWAAKOBA BBE MU BIMU KU BIBUUZO EBIMULEKAMU:

***TERMINATION CLAUSE (IF THEY ANSWER NO TO ANY OF THE INCLUSION QUESTIONS:***

Webale kubaagho okuba nga tusobola okwogeraku niighe; aye olwa leero,titwiidha kweyongera kukubuuza bibuuzo bindi kubanga***(Insert reason depending on which inclusion question was given a no response) Thank you for availing yourself so that we can talk to you; but for today we will not ask you any more questions because of (Insert reason depending on which inclusion question was given a no response)***

**CONSENT**

**This is to be used together with the Informed consent form and information sheet for patients and caregivers**

| - Amainha gange nhinze__________________ndi kukolera eitendekero lya Makerere university   *My name is __________. I am working for Makerere University* |
| --- |
| - Tuli kukola okunoonereza kuno okwega ku ndowoozayo ku mpeereza y’obwidhandabi obusookerwaku.   *We are conducting this study to learn about your perception on the delivery of primary health care services.* |
| - Tuli kukyaalira amalwaliro munaana okuva mu Iganga era tuli kubuuza abalwaire abawera amakumi asatu mu buli irwaliro.   *We are visiting 8 facilities across Iganga and are interviewing up to thirty patients in each facility.* |
| - Buli mulwaire tuli kubuuza ebibuuzo ebigema empeereza yebafuna, enkolagana n’abakola mu by’obulamusawo n’okusobola obulamu bwaibwe.   *For each patient we are asking questions concerning services they received, their interaction with health care workers and management of their health*. |
| - Okubuuza woonawoona kwiidha kutwala edhakika makumi asatu   *The whole interview will take approximately 30 minutes.* |
| - Titwetaaga kumanha mainhago, era amawulire goonagoona gonatugha gaidha kutwalibwa nga ga kyaama.   *We do not need to know your name, and all information you give us will be treated confidentially.* |
| - Okwenhigira mu kunoonereza kuno kwa kyeyendeire. Bwoikiriza buti me oluvainhuma n’okyuusa ekirowooo, oli waidembe okuvamu esaawa yoonayoona.   *Your participation in this study is voluntary. If you agree now and later change your mind, you are free to withdraw at any time.* |
| - Tubite mu lupapula olusaba olukusa   *Go through the ICF form*   - Otegeire? Olinaku ebibuuzo byonabyona?   *Have you understood? Do you have any questions?* |
| - Kirungi okuja mu maiso n’okubuuza?   *Is it okay that we proceed with the interview?(circle one appropriate response)*  Yes................................. 1  No.................................. 2 |

| 1.5 | OLUKUSA: Nkakasa nti nsomyemu olupapula oluliku obubaka era n’olupapula olusaba olukusa era nainonola okunoonereza kunoeri abuuzibwa nti era ategeera engeri n’omugaso gw’okunoonereza era yaikiriza okwenhigiramu. Awereibwa omukisa okubuuza ebibuuzo ebiiribwamu mu bumativu. Kopi y’ekiwandiiko ekisaba olukusa esigaire n’abuuzibwa n’endagiriro olw’ebibuuzo by’ayinza okuba nanbyo.  *CONSENT: I certify that I have gone through the information sheet and consent form and explained this study to the interviewee and that s/he understands the nature and purpose of the study and consents to participate. S/he has been given an opportunity to ask questions which have been answered satisfactorily. A copy of the consent form has been left with the interviewee with contacts for any questions they may have* | YES ……………………………………………. 1  NO ………………..……………………………. 2  Patient signature or thumbprint |
| --- | --- | --- |
| 1.6 | Esaawa okubuuza yekutandiikireku Time Interview Started. ………………… | HOUR[__][__] MINUTE[__][__] |

**MAIN QUESTIONS(Circle all appropriate responses)**

| Buti nandienze okubuuzaku ebibuuzo bitono ku ighe n’amakaago  ***Now I would like to ask a few questions about you and your family. (circle appropriate response(s))*** | | |
| --- | --- | --- |
| 2.0 | Musaadha oba Mukazi  ***Male or Female*** | Musaadha  Male………………………….................... 1  Mukazi  Female………………………………......... 2 |
| 2.1 | Olina emyaaka emeka?  ***How old are you?*** | WAGATI W’EMYAAKA 16 KU 24…......1  BETWEEN 16-24YRS…............................1  WAGATI W’EMYAAKA 25 KU 44..........2  BETWEEN 25-44YRS…............................2  EMYAAKA 45 N’OKWIIRA WAIGULU..3  45YRS & ABOVE ……..............................3  TIIDHI........…………;;…….…................99  DK ………………………….…................99 |
| 2.2 | Wamaliriza okusoma kwa pulaimale?  ***Did you complete primary school education?*** | YII/YES………………………...........................1  MBE/NO…. ………………………...................2 |
| 2.3 | Wamaliriza okusoma kwa siniya?  ***Did you complete secondary school education?*** | YII/YES………………………...........................1  MBE/NO…. ………………………...................2 |
| 2.4 | Osobola okusoma embaluwa eghandikiibwa mu luzungu?  ***Can you read a letter written in English?*** | YII/YES………………………...........................1  MBE/NO…. ………………………...................2 |

| 2.5 | | Lwaki oidhe okukyaalira eirwaliro olwa leero?***Why did you visit the facility today?***   1. Mweene mulwaire   ***SICK THEMSELVES***   1. Kuleeta mwaana mulwaire   *BRINGING SICK CHILD(<16 YRS )*   1. Kuleeta mulwaire mukulu   *BRINGING SICK ADULT*   1. Kwidhandaba maama na mwaana   *MCH*   1. Entegeka y’eizaire (FP)   Family planning  *FP*   1. Kunwa bulezi/kukeberebwa nga mmaze okuzaala   *ANC/PNC*   1. Akalwaliro akalimu buli kimu ku ndabirira ey’abalwaire   *ART clinic*   1. *Kukeberebwa/kuwandikirwa*   *EXAMINATION/ PRESCRIPTION*   1. *Ekindi OTHER (SPECIFY)* | | YII/*YES* MBE/*NO*  1 2  1 2  1 2  1 2  1 2  1 2  1 2  1 2  1 2  ________________________________________ | | |
| --- | --- | --- | --- | --- | --- | --- |
| 2.6 | | Gunno n’omulundi gwo ogusooka okwidha kwiirwaliro?  ***Is this your first visit to the facility?*** | | YII/YES……………………….................... 1  MBE/NO…........………….......................... 2 | | |
| 2.7 | | Wano n’ekifo ekisinga kuba kya kumpi mu waidhandabirwa awasooka?  ***Is this the nearest primary care facility to your area of residence?*** | | YII/YES……………………….................... 1  MBE/NO…........………….......................... 2 | | |
| 2.8 | | Lwaki wasazeewo okwiidha ku irwaliro lino?  ***Why did you choose to come to this facility?*** *(select all that apply)*? *(Londaku ebyo byoonabyoona ebikola)* | | N’eriri okumpi ni waka ewange  ***It is the nearest to my home………….. 1***  Lwa bwerere  ***It is free ………………………………. 2***  Obulungi bw’omutindo ogw’empeereza  ***Good quality of care experience ……... 3***  Nnenda abasawo  ***I like the clinician(s) …………………. 4***  Bansindika wano  ***I was referred here …………………… 5*** | | |
| 2.9 | | Kikutwaliire ibanga ki (mu saawa) okwidha ku irwaliro era na ntambulaki?  ***How long (in hours) did it take you to come to the facility and by which means?*** | |  | | |
| Buti nandienze okubuuzaku ebibuzo ebigema ku gha w’ofuna obwidhandhabi  ***Now I would like to ask you some questions concerning where you get health care*** | | | | | | |
| 2.10 | | Bwoba n’ekizibu ku bulamu, ogyaawa?  ***When you have a new health problem, where do you go?*** | | Akalwaliro akasokerwaku***……............................. 1***  ***The primary health care clinic***  Akaduuka akatunda eiddagala***………………….. 2***  ***The local drug shop***  Omwidhandabi owo ku kitundu***………………... 3***  ***The community health worker***  Eirwaliro eikulu mu disitulikiti***…………….…….. 4***  ***The district hospital***  Ekindi (inhonola)…………………………………5  ***Other specify*** | | |
| 2.11 | | Ogya bakukeberaku?  Do you go for medical check-ups? | | YII/YES………………………..........................1  Inhonola gha..................................................  Specify where  MBE/NO……......………….......................... 2 | | |
| 2.12 | | Bwobona omukugu, omusawowo ateekwa okukakasa oba okuwandikira ebaluwa okwongerayo?  ***When you see a specialist, does your doctor have to approve or give you referral?*** | | YII/YES……………………….................... 1  MBE/NO……......………….......................... 2 | | |
| 2.13 | | Omusawo aghandika akabaluwa eri omukugu gwebaba bakukobye?  ***Does the clinician write a note to the recommended specialist?*** | | YII/YES……………………….................... 1  MBE/NO…........………….......................... 2 | | |
| 2.14 | | Ebizuulibwa okuva mu kukyaalira omukugu mubyogeraku n’omusawo wo asokerwaku?  ***Do you discuss the findings from the visit to the specialist with your primary care clinician?*** | | YII/YES……………………….................... 1  MBE/NO…........………….......................... 2 | | |
| ***Buti nandienze okubuuzaku ebibuuzo ebigema ku irwaliro lino. Oyinza obutamanha kya kwiiramu ku byonabyona-otaloba kunkobera eyo bweeba nga n’ensonga. Now I would like to ask you some questions concerning this facility. You might not know all of the answers - do not hesitate to let me know if that is the case.(circle appropriate response)*** | | | | | | |
|  |  | | Olunaku *Day* | | Yii/Yes Mbe/No | Esaawa (nga Ibiri edhokunkyo okutuuka ku Ikumi nandala edh’eiggulo)  Time (e.g. 8am-5pm) |
| 3.0 | Oidhi oba nga eirwaliro liigulwa era saawa imeka mu biseera bino?  ***Do you know if and when the facility is open on:*** | | Ennaku edh’okukola *Weekdays* | | 1 2 |  |
|  |  |  | Weekendi *Weekends* | | 1 2 |  |
|  |  |  | Obwiire  *At night* | | 1 2 |  |
|  |  |  | Ennaku enkulu *Public holidays* | | 1 2 |  |
| 3.1 | Bw’olwala, nga eirwaliro liigule, omuntu ow’agho asobola okukubona ku lunaku lweene olwo?  ***When you get sick, and the facility is open, would someone from there see you the same day?*** | | YII/YES……………………….................... 1  MBE/NO…........………….......................... 2 | | | |
| 3.2 | Bw’olwala, nga eirwaliro liigule, obona omusawo n’omulala oyo buli mulundi?  ***When you get sick, and the facility is open, do you see the same clinician every time?*** | | YII/*YES*……………………….................... 1  MBE/*NO*…........………….......................... 2 | | | |
| 3.3 | Ekifo bwewaba waigale,eriyo ennamba y’eisimu kw’osobola okukuba bwoba olwaire?  ***When the office is closed, is there a phone number you can call when you get sick?*** | | YII/YES……………………….................... 1  MBE/NO……. ………………...................... 2  TAIDHI/DK…………………...................... 99 | | | |
| 3.4 | Bwolwala nga eirwaliro liigale, omuntu ow’agho asobola okukubona ku lunaku lweene olwo?  ***When the facility is closed and you get sick, would someone there see you the same day?*** | | YII/YES……………………….................... 1  MBE/NO……. ………………...................... 2  TAIDHI/DK…………………...................... 99 | | | |
| 3.5 | Osobola okutuukirira/okusaba omusawo gwoyenda ku irwaliro?  ***Are you able to access/request a specific clinician at the facility?*** | | YII/*YES*……………………….................... 1  MBE/*NO*…........………….......................... 2 | | | |
| 3.6 | Osobola okutuukirira omusawo gwoyenda ku issimu?  ***Are you able to access a specific clinician by phone?*** | | YII/*YES*……………………….................... 1  MBE/*NO*…........………….......................... 2 | | | |
| 3.7 | Waali owulireku ebibiina ebiwanirira abalwaire?  ***Have you ever heard of any patient support groups?*** | | YII/*YES*……………………........................ 1  Inhonola gha / *If yes specify*………………….  MBE/*NO*……. …………………….................2 | | | |
| 3.8 | Oidhiku omuntu yenayena oba omukiise mu bibiina ebiwanirira abalwaire?  ***Do you know any patient support groups members or representatives?*** | | YII/*YES*……………………….................... 1  MBE/*NO*…........………….......................... 2 | | | |

| Buti nandienze okubuuzaku ebibuuzo ebitono ku byobiseemu wano ku irwaliro olwa leero.  ***Now I would like to ask a few questions about your experience at the facility today (circle all appropriate responses)*** | | | | | | |
| --- | --- | --- | --- | --- | --- | --- |
| 4.0 | Ani gwemuwayiiya naye ku irwaliro olwa leero?  ***Who did you interact with at the facility today? (insert number of staff)*** | Waiting bay__________________________________  Registration__________________________________  Consultation room_____________________________  Laboratory___________________________________  Drug dispenser________________________________  Other area____________________________________ | | | | |
| 4.1 | Olinze kumala ibangaki nga okaali:  ***How long did you wait before:***  ***(Wandiika omuwendo gwa saawa n’edakiika mu kifo ekitereibwaawo) (Write the number of hours and minutes in the space provided)*** | Kubona dokita.........................[__] Hours [__] Minutes  ***Seeing the doctor***  Kufuna biviire mu kukebera…[__] Hours [__] Minutes***Getting lab results***  Kufuna idagala.……..............[__] Hours [__] Minutes  ***Getting medication***  Omugaite gw’ekiseera ku irwaliro[__] Hours [__]***Minutes***  ***Total time at the facility*** | | | | |
| Mu kino kitundu, nandienze okwiizaku einhuma ku kuwayakwo n’omusawo leero era ote ebigambo bino wammanga ku idaalaokuva ku kwiikiririza irala okutuuka ku butaikiririza irala.***For this section, I would like you to look back at your interaction with the clinician today and rate the following statements ranging from strongly agree to strongly disagree (circle one appropriate response)***  **Sources:***(Mead and Bower 2002, Stewart 2003, Hudon, Fortin et al. 2011)* | | | | | | |
| **4.2** Okwekenenia obulamu, obulwaire, ni by’obyitamu mu kulwala: Omutendera ogw’okwebuzaaku  ***Exploring health, disease and the illness experience: Consultation process*** | | **Ndikiririza irala Strongly Agree** | **Ndikiriza Agree** | **Tasalawo/ali awo wagati Undecided**  **/ Neutral** | **Taikiriza Disagree** | **Taikiririza irala Strongly Disagree** |
| Omusawo anamwisa mu ngeri endeteire okuwulira obulungi  ***The health worker greeted me in a way that made me feel comfortable*** | | 1 | 2 | 3 | 4 | 5 |
| Omusawo akozeisa olulimi lwensobola okutegeera  ***The health worker used a language that I could understand*** | | 1 | 2 | 3 | 4 | 5 |
| Ekifo aweebuuzibwaamu kibaire kya kyaama era nga kirungi  ***The consultation space was private and comfortable*** | | 1 | 2 | 3 | 4 | 5 |
| Omusawo ankubiriza okugha ebirowoozo byange ebigema ku bulamu/bulwaire bwange.  ***The health worker encouraged me to express my thoughts concerning my health/illness*** | | 1 | 2 | 3 | 4 | 5 |
| Nsoboire okwogera ku nsonga dhange edhindeese olwa leero  ***I was able to discuss my reasons for coming today*** | | 1 | 2 | 3 | 4 | 5 |
| Nkubagainia ebirowoozo lwaki kibaire kya mugaso nze okwiidha ku irwaliro (nga obutasigala waka oba obutagya ku musawo wa kirugavu)  ***I discussed why it was important for me to come to the facility (i.e. and not stay at home or visit chemist…)*** | | 1 | 2 | 3 | 4 | 5 |
| Omusawo abaire wa kisa  ***The health worker was sympathetic*** | | 1 | 2 | 3 | 4 | 5 |
| Omusawo awuliriza bulungyi era kyendowoziiza nti n’embeera/ekizibukimunoonie.  ***The health worker listened carefully and was interested in what I thought the situation / problem was*** | | 1 | 2 | 3 | 4 | 5 |
| Nsoboire okwinhonola obubonero bwange  ***I was able to describe my symptoms*** | | 1 | 2 | 3 | 4 | 5 |
| Omusawo awuliriza bulungyi erakimunoonie bwendogeire ku bubonero bwange.  ***The health worker listened carefully and was interested when I talked about my symptoms*** | | 1 | 2 | 3 | 4 | 5 |
| Omusawo ambuziiza ku ki kyendowooza nga n’ekiri kuviiraku obubonero bwange.***The health worker asked me what I believe is causing my medical symptoms*** | | 1 | 2 | 3 | 4 | 5 |
| Omusawo ayenze inho okumanha obwidhandabi bwenafunaku einhuma  ***The health worker was interested in what treatment I had before*** | | 1 | 2 | 3 | 4 | 5 |
| Nsoboire okwinhonolaowbidhandhabi bwenabaire nfunieku e’inhuma  ***I was able to explain treatment that I had got before*** | | 1 | 2 | 3 | 4 | 5 |
| Omusawo asoboire okulinga mu biwandiiko byange eby’obulamu era yambuuza ebibuuzo  ***The health worker was able to look back at my health records and ask questions*** | | 1 | 2 | 3 | 4 | 5 |
| Omusawo ayenze inho okumanha ki kyenayenze kikolebwe  ***The health worker was interested in what I wanted to be done*** | | 1 | 2 | 3 | 4 | 5 |
| Ndizeemu ebibuuzo by’omusawo byonabyona mu bulambulukufu  ***I answered all the health worker’s questions honestly*** | | 1 | 2 | 3 | 4 | 5 |
| Omusawo ategeire kyembaire ow’okwogera  ***The health worker understood what I had to say*** | | 1 | 2 | 3 | 4 | 5 |
| **4.3 Okwekeeneenia n’endidhandaba**  ***Diagnosis and treatment***  ***(circle one appropriate response)***  *Sources: (Mead and Bower 2002, Stewart 2003, Hudon, Fortin et al. 2011)* | | **Ndikiririza irala Strongly Agree** | **Ndikiriza Agree** | **Tasalawo/ali awo wagati Undecided**  **/ Neutral** | **Taikiriza Disagree** | **Taikiririza irala Strongly Disagree** |
| Singa kyetagisa okukebera omubiri, omusawo ainhonoire bulungi ekikoleibwa na lwaki?  ***If a physical examination was required, the health worker fully explained what was done and why*** | | 1 | 2 | 3 | 4 | 5 |
| Singa kyetagisa,omusawo akwinhonoire okukeberwa okwetagisa okuzuula ekizibu  ***If required the health worker explained the lab tests needed to explore the problem*** | | 1 | 2 | 3 | 4 | 5 |
| Omusawo ainhonoire ebiviire mu kukeberebwa  ***The health worker explained the results of the lab tests*** | | 1 | 2 | 3 | 4 | 5 |
| Omusawo ainhonoire ekizibu n’ekirigha  ***The health worker explained what the problem was*** | | 1 | 2 | 3 | 4 | 5 |
| Omusawo ainhonoire kiki (biki)ebivaaku ekizibu  ***The health worker explained what the cause(s) of the problem was (were)*** | | 1 | 2 | 3 | 4 | 5 |
| Omusawo atesiiza ninzeeby’okulondaku ku ndidhandaba  ***The health worker discussed treatment options with me*** | | 1 | 2 | 3 | 4 | 5 |
| Omusawo ankobeire egya okuba endhidandaba /obwidhandabi  ***The health worker told me what the treatment / medication would do*** | | 1 | 2 | 3 | 4 | 5 |
| Omusawo ainhonoire obuzibu obuyinza okuva mu ndhidandaba /obwidhandabi  ***The health worker explained treatment/ medication side effects*** | | 1 | 2 | 3 | 4 | 5 |
| Omusawo ampaire amawulire agamala nga bwenkyenze  ***The health worker gave me as much information as I wanted*** | | 1 | 2 | 3 | 4 | 5 |
| Omusawo ankubiriza okubuuza ebibuuzo  ***The health worker encouraged me to ask questions*** | | 1 | 2 | 3 | 4 | 5 |
| Mpuliire bulungi okubuuza ebibuuzo  ***I felt comfortable to ask questions*** | | 1 | 2 | 3 | 4 | 5 |
| Omusawo aizemu ebibuuzo n’ensonga dhange  ***The health worker responded to my questions and concerns*** | | 1 | 2 | 3 | 4 | 5 |
| Tukubagainia ebirowoozo n’omusawo ku mitendera egyiiraku, nga mwotaire okulondoola n’okukyaala okwiiraku  ***The health worker discussed next steps, including follow-up plans and next visits*** | | 1 | 2 | 3 | 4 | 5 |
| Omusawo akebeire okukakasa nga ntegeire buli kintu  ***The health worker checked to be sure I understood everything*** | | 1 | 2 | 3 | 4 | 5 |
| Omusawo akebeire okukakasa nga entegeka y’obwidhandabi nnangu gyendi  ***The health worker checked to be sure the treatment plan was manageable for me*** | | 1 | 2 | 3 | 4 | 5 |
| Omusawo ainhonoire ebiyinza okuva mu mu kizibu/mbeera ey’obulamu bwange yebwiidhaku  ***The health worker explained the long term consequences of my medical problem / condition*** | | 1 | 2 | 3 | 4 | 5 |
| The health worker treated me with respect | | 1 | 2 | 3 | 4 | 5 |
| **4.4 Okutegeera omuntu yenayena: Ekisa, obwesigwa, era n’ekinonereze kyekita ku bulamu**  ***Understanding the whole person: Empathy, trust and interest of effect on life***  ***(circle one appropriate response)***  *Sources: (Mead and Bower 2002, Stewart 2003, Hudon, Fortin et al. 2011)* | | **Ndikiririza irala Strongly Agree** | **Ndikiriza Agree** | **Tasalawo/ali awo wagati Undecided**  **/ Neutral** | **Taikiriza Disagree** | **Taikiririza irala Strongly Disagree** |
| Omusawoyalaze nga anfaakuera yandabirira nga omuntu  ***The health worker showed care and concern about me as a person*** | | 1 | 2 | 3 | 4 | 5 |
| Omusawo mutairemu ekitiibwa era na mwiikiriza nga omuntu  ***I showed the health worker respect and accepted them as a person*** | | 1 | 2 | 3 | 4 | 5 |
| Omusawo andeteire okuwulira obutebenkevu  ***The health worker made me feel at ease*** | | 1 | 2 | 3 | 4 | 5 |
| Omusawo alinze inho kubuzibu obulwaire/embeera bweeta ku maka gange oba ku bulamu bwange  ***The health worker was interested in the effect of the problem/condition on my family or personal life*** | | 1 | 2 | 3 | 4 | 5 |
| Omusawo alinze inho kubuzibu obulwaire/embeera bweeta ku mirimu gyange egya buliidho  ***The health worker was interested in the effect of the problem/condition on everyday activities*** | | 1 | 2 | 3 | 4 | 5 |
| Omusawo aikiriza ku li ekizibu lwekirimalwaagho oba okwiikaikanizibwa  ***The health worker was positive about when the problem would be solved or settled*** | | 1 | 2 | 3 | 4 | 5 |
| Omusawo antaamu ekitiibwa  ***The health worker respects me*** | | 1 | 2 | 3 | 4 | 5 |
| Nnesiga omusawo okukola okusalawo okulungi ku ndabirira ey’obulamu bwange  ***I trust the health worker to make good decisions about my health care*** | | 1 | 2 | 3 | 4 | 5 |
| Omusawo ono afa inho ku muwendo okusingaku n’ogwo ogwetagibwa ku bulamu bwange  ***This health worker cares more about the cost than what is needed for my health*** | | 1 | 2 | 3 | 4 | 5 |
| Omusawo ono ankoberanga amazima ku bulamu bwange, ni bwekiba nti amawulire mabi  ***This health worker would always tell me the truth about my health, even if it was bad news*** | | 1 | 2 | 3 | 4 | 5 |
| Singa ensobi yakolebwa mu idagala lyange, omusawo wange agezaaku okunkweka ensobi  ***If a mistake was made in my treatment, my health worker would try to hide it from me*** | | 1 | 2 | 3 | 4 | 5 |
| **4.5 Obwesigwa n’obumativubw’omulwaire Patient confidence and satisfaction**  ***(circle one appropriate response)***  *(Mead and Bower 2002, Stewart 2003, Van Eygen, Van Lerberghe et al. 2007, Hudon, Fortin et al. 2011)* | | **Ndikiririza irala**  ***Very confident*** | **Ndikiriza Agree**  ***Somewhat confident*** | **Tasalawo/ali awo wagati *Undecided***  ***/ Neutral*** | **Taikiriza *Not confident*** | **Taikiririza irala**  ***Not confident at all*** |
| Ndi mumativu inho n’okukyalira kwange eri omusawo  ***I am totally satisfied with my visit to the health worker*** | | 1 | 2 | 3 | 4 | 5 |
| Omusawo ono kwaga asobola okukola ku buli kizibu kyonakyona eky’obulamu kyenhinza okuba nakyo  ***This health worker can take care of almost any medical problem I might have*** | | 1 | 2 | 3 | 4 | 5 |
| Nsobola okwidha eri omusawo ono okuyambibwa mu kizibu ekigema ku nze oba ku mbeera ey’obwongo  ***I could go to this health worker for help with a personal or emotional problem*** | | 1 | 2 | 3 | 4 | 5 |
| Ndi n’okwiikiriza nti omusawo andidhi n’ebyafaayo byange  ***I’m confident that the health worker knows me and my history*** | | 1 | 2 | 3 | 4 | 5 |
| Ndi mwekakafu nti omusawo ategeera ebirowoozo byange  ***I’m confident that the health worker understands my emotion needs*** | | 1 | 2 | 3 | 4 | 5 |
| Ndi n’okwiikiriza nti omusawo ategeera okusalagho kwange mu by’obughangwa n’omwoyo  ***I’m confident that the health worker understands my cultural and spiritual preferences*** | | 1 | 2 | 3 | 4 | 5 |
| Ndi n’okwiikiriza nti omusawo aidhi ku buvunanhizibwa bwange mu maka, ku mulimu oba ku issomero  ***I’m confident that the health worker knows about my responsibilities at home, work or school*** | | 1 | 2 | 3 | 4 | 5 |
| Ndi mutebenkevu okubuuza ebibuuzo ebingemaku  ***I’m comfortable asking personal questions*** | | 1 | 2 | 3 | 4 | 5 |
| Ntegeera obwidhandabi/entegeka ey’eidagala  ***I understand the treatment / medical plan*** | | 1 | 2 | 3 | 4 | 5 |
| Nsobola okwinhonola eidagala lyendikumira  ***I can explain the medicines I am taking*** | | 1 | 2 | 3 | 4 | 5 |
| Ntegeera ebiragiro eby’omusawo  ***I understand the health worker’s directions*** | | 1 | 2 | 3 | 4 | 5 |
| Nnina enkyuukakyuuka ennungi yenesubira mu bulamu bwange  ***I have a good idea about the changes to expect in my health*** | | 1 | 2 | 3 | 4 | 5 |
| Nsobola okutegeera engeri obwidhandabi bwange gyebuli kutambula  ***I am able to understand how my treatment is going*** | | 1 | 2 | 3 | 4 | 5 |
| Mpulira nga omusawo ono ambisiiza bundi olw’e ighanga lyange.  I feel this health worker treated me differently because of my ethnicity | | 1 | 2 | 3 | 4 | 5 |
| Mpulira nga omusawo ono ambisiiza mu ngeri ey’endhawulo olw’obwegerese bwange  ***I feel this health worker treated me differently because of my level of education*** | | 1 | 2 | 3 | 4 | 5 |
| Mpulira nga omusawo ono ambisiiza mu ngeri ya ndawulo olw’obusobozi bwange mu kusasula  ***I feel this health worker treated me differently because of my ability to pay*** | | 1 | 2 | 3 | 4 | 5 |
| **Okwegeresa n’okutumbula eby’obulamu Health education and promotion**  ***(circle one appropriate response)***  **Sources:***(Mead and Bower 2002, Stewart 2003, Hudon, Fortin et al. 2011)* | | **Ndikiririza irala Strongly Agree** | **Ndikiriza Agree** | **Tasalawo/ali awo wagati Undecided**  **/ Neutral** | **Taikiriza Disagree** | **Taikiririza irala Strongly Disagree** |
| Omusawo ayogeire ku ngeri edh’okukendeeza ku mikisa egy’okufuna obulwaire yebwiidha  ***The health worker talked about ways to lower the risk of future illness*** | | 1 | 2 | 3 | 4 | 5 |
| Ampaire amagezi ku ngeri ey’okuziyizaamu ebizibu eby’obulamu mu yebwiidha (sukaali, emizze emirungi mu bulamu, okukola duyiro, okwegandaga okukalamu nga kigiira ku bulwaire oba embeera…)  ***Advised me how to prevent future health problems (diet, health habits mentioned exercise, safe sex as relevant to illness or condition…)*** | | 1 | 2 | 3 | 4 | 5 |
| **4.6** Okwongerayokw’okulabirira  ***Continuity of care: referrals***  ***(circle one appropriate response)***  **Sources:** *(Mead and Bower 2002, Stewart 2003, Hudon, Fortin et al. 2011)* | | **Ndikiririza irala Strongly Agree** | **Ndikiriza Agree** | **Tasalawo/ali awo wagati Undecided**  **/ Neutral** | **Taikiriza Disagree** | **Taikiririza irala Strongly Disagree** |
| Omusawo ono aidhi li okukeberebwa kwange lwekutuuka  ***This health worker knows when I am due for a check up*** | | 1 | 2 | 3 | 4 | 5 |
| Omusawo ono buli kiseera alondoola ekizibu kyenali nakyo bwenkyaala ku mulundi ogwiiraku oba akuba eisimu  ***This health worker always follows up on a problem I had at the next visit or by phone*** | | 1 | 2 | 3 | 4 | 5 |
| Omusawo ono buli kiseera anondoola ku kukyaala okwiiraku n’abasawo abandi  ***This health worker always follows up on the next visit with other health workers*** | | 1 | 2 | 3 | 4 | 5 |
| This health worker tells me when I need to see a specialist | | 1 | 2 | 3 | 4 | 5 |
| Omusawo ono anamba okukola obulagane bwemba nnenda okubona omukugu/omusawo ku irwaliro erririku waigulu  ***This health worker helps me to book appointments when I need to see a specialist/ health worker in a higher level facility*** | | 1 | 2 | 3 | 4 | 5 |
| Omusawo ono ayogera n’abasawo bale abandi bembona  ***This health worker communicates with the other health providers I see*** | | 1 | 2 | 3 | 4 | 5 |
| Omusawo ono aidhi ebyaviire mu kukeberebwa bwenakyaalire omukugu  ***This health worker knows what the results of the specialist visit were*** | | 1 | 2 | 3 | 4 | 5 |
| Nga maze okukyaalira omukugu oba okufuna empereza ey’endhawulo, omusawo ono ayogeire niiwe ku kyabaire mu kukyaala  ***After going to the specialist or special service, this health worker talked with you about what happened at the visit*** | | 1 | 2 | 3 | 4 | 5 |
| Omusawo wo aboneike nga ayenze inho omutindo ogw’empereza gw’ofuna okuva ku mukugu oyo oba empereza ey’endhawulo  ***Your health worker seemed interested in the quality of care you get from that specialist or special service*** | | 1 | 2 | 3 | 4 | 5 |
| **4.7** Okukola okusalawo okw’aghalala  **Joint Decision Making**  ***(circle one appropriate response)***  **Sources:** *(Mead and Bower 2002, Stewart 2003, Hudon, Fortin et al. 2011)* | | **Ndikiririza irala Strongly Agree** | **Ndikiriza Agree** | **Tasalawo/ali awo wagati Undecided**  **/ Neutral** | **Taikiriza Disagree** | **Taikiririza irala Strongly Disagree** |
| Omusawo yanhigire mu kusalawo nga bwenakyenze  ***The health worker involved me in decisions as much as I wanted*** | | 1 | 2 | 3 | 4 | 5 |
| Twogeire era twembi twaikirizagania ku kizibu n’ekirigha  ***We discussed and together agreed on what the problem was*** | | 1 | 2 | 3 | 4 | 5 |
| Tuviireyo n’ekigendererwa eky’obwidhandabi/enteekateeka y’obwidhandabi  ***We came up with the goals of treatment / health care plan*** | | 1 | 2 | 3 | 4 | 5 |
| Tutesiiza ku mitendera egyiiraku omuli n’entegeka edh’okundutaku  ***We discussed next steps, including any follow-up plans*** | | 1 | 2 | 3 | 4 | 5 |
| Tutesiiza ku buvananhizibwa bwa buli omu (omusawo n’omulwaire) mu ntegeka yange ey’obwidhandabi  ***We discussed our respective roles (the health worker and the patient) in my health care plan*** | | 1 | 2 | 3 | 4 | 5 |
| Omusawo ampaire amawulire gonagona genenze  ***The health worker gave me all the information I need*** | | 1 | 2 | 3 | 4 | 5 |
| Ekiseera kyensoboire okumala n’omusawo ono tikibaire kinene kimala kukola ku buli kimu kyenhenze  ***The time I was able to spend with this nurse was not long enough to deal with everything I wanted*** | |  |  |  |  |  |
| Mpaire endowooza yange (okwikiriza oba obutaikiriza) ku bika by’okukebera oba obwidhandabi omusawo wange byalagiire  ***I gave my opinion (agreement or disagreement) about the types of tests or treatment that my health worker ordered*** | | 1 | 2 | 3 | 4 | 5 |
| Omusawo yagemagainia okubusabuusa kwonakwona ku kukebera oba obwidhandabi ebyasalibwaawo  ***The health worker handled any doubts about the tests or treatment that were recommended*** | | 1 | 2 | 3 | 4 | 5 |
| Omusawo yankubiriza okutwaala obuvunanhizibwa bwenenda mu kulabirirwa kwange  ***The health worker encouraged me to take the role I wanted in my own care*** | | 1 | 2 | 3 | 4 | 5 |
| Omusawo akuleka waalinga ku biwandiikobyo eby’obulamu  ***The health worker lets you look at your medical records*** | | 1 | 2 | 3 | 4 | 5 |
| **4.8** Ebiva mu kwidhandabwa: mu kitundu kino, nandienze olowoze ku ngeri obumanhirivubwo ku irwaliro gyebukoze ku bulamubwo.  **Outcomes of care: *for this section, I would like you to think about how your experience at the facility has affected your health(circle one appropriate response)*** | | | | | | |
| **Sources:** *(Mead and Bower 2002, Stewart 2003, Hudon, Fortin et al. 2011)* | | **Ndikiririza irala Strongly Agree** | **Ndikiriza Agree** | **Tasalawo/ali awo wagati Undecided**  **/ Neutral** | **Taikiriza Disagree** | **Taikiririza irala Strongly Disagree** |
| Obubonero bwange n’obulumi bikendeire  ***My symptoms and pain have reduced*** | | 1 | 2 | 3 | 4 | 5 |
| Kikendeziiza okutya kwange n’okweralikirira  ***It has lessened my fears and anxiety*** | | 1 | 2 | 3 | 4 | 5 |
| Nsobola okwira ku mirimu gyange egya buliidho  ***I am able to return back to my routine activities*** | | 1 | 2 | 3 | 4 | 5 |
| Nsobola okubaaku nikyenkola singa embeera y’obulamu bwange eyononekamuuku (ndidi ow’okutukirira, eky’okukola)  ***I am able to react if my health deteriorates***  ***(I know who to contact, what to do)*** | | 1 | 2 | 3 | 4 | 5 |
| Ndimu amaani okugiira ku idagala eriwandiikibwa  ***I am motivated to follow the treatment prescribed*** | | 1 | 2 | 3 | 4 | 5 |
| ***Which advice do you find difficult to follow?*** | |  | | | | |
| Ekisobozesa omulwaire  ***Patient enablement instrument*(Van Eygen, Van Lerberghe et al. 2007)** | | Okusinziira ku kukyaalakwo eri omusawo leero, owulira nga oli?  ***As a result of your visit to the nurse today do you feel you are?*** | | | | |
|  |  | Bulungi inho  ***Much better*** | Bulungiku  ***better*** | **N’ekirala**  **same** | N’ekirala oba katono  ***Same or less*** | Ghazira waire  ***Not at all*** |
| Osobola okugumira obulamu?  ***Able to cope with life?*** | | 1 | 2 | 3 | 4 | 5 |
| Osobola okutegeera obulwairebwo?  ***Able to understand your illness?*** | | 1 | 2 | 3 | 4 | 5 |
| Osobola okugumira obulwairebwo?  ***Able to cope with your illness?*** | | 1 | 2 | 3 | 4 | 5 |
| Osobola okwekuuma nga oli mulamu?  ***Able to keep yourself healthy?*** | | 1 | 2 | 3 | 4 | 5 |
| Oli mwekakafu ku bulamubwo?  ***Confident about your health?*** | | 1 | 2 | 3 | 4 | 5 |
| Osobola okweyamba?  ***Able to help yourself?*** | | 1 | 2 | 3 | 4 | 5 |
| Overall satisfaction | | | | | | |
|  | | ***Very satisfied*** | ***satisfied*** | ***indifferent*** | ***Not satisfied*** | ***Not satisfied at all*** |
| Number of health workers | |  |  |  |  |  |
| How health workers communicated with you | |  |  |  |  |  |
| Your treatment / health plan? | |  |  |  |  |  |
| Advice / follow-up | |  |  |  |  |  |
| Will you come back to this facility | | Yes | | No | |  |
| Would you like to see the same health worker on your next visit? | | Yes | | No | |  |
| Would you refer your friend or family to this facility | | Yes | | No | |  |

| 7.0 | Additional questions from the pilot | | |
| --- | --- | --- | --- |
|  | What enables you to participate in discussions and decisions reached together with their health provider? |  | |
|  | What hinders you from participating in discussions and decisions reached together with their health provider? |  | |
|  | Are you a member of any patient support group or organisation? (if yes specify name and type of group of patients) |  | |
|  | During your visit to the facility today did you feel like you were handled differently because of your: circle appropriate response | | |
|  |  | YES | NO |
|  | Condition or illness | 1 | 2 |
|  | Age | 1 | 2 |
|  | Gender | 1 | 2 |
|  | Education | 1 | 2 |
|  | Religion | 1 | 2 |
|  | Ethnicity | 1 | 2 |
|  | Financial ability to pay for the services at the health care facility | 1 | 2 |
|  | If treated differently, what was done differently, why do they feel they were treated differently |  | |
|  | Anoonereza ate wano byayogeraku/byaboine ku kubuuza kuno  ***Field worker to enter any comments/ observations about this interview*** |  | |

Webale inho olw’okwenhigirakwo mu kubuuzibwa kuno. Olinaku ebibuuzo ebindi?

***Thank you very much for your participation in this interview. Do have any questions***?

INTERVIEW ENDED AT HR [__][__] MIN [__][__]

**CHECKED BY:**INTERVIEWER CODE [__][__] DATE ___________

**INTERVIEW GUIDE FOR IN DEPTH INTERVIEWS WITH PATIENTS**

**Study title: Understanding patient-centred care approaches at the level of primary health care facilities in sub-Saharan Africa: the case of Uganda**

Thank you for welcoming us into your home. As we had introduced ourselves during your visit to the facility, my name is _______________and this is my colleague _________________ and we are working together with Makerere university looking into the relationships between patients and health workers and how to improve the quality of care that you receive at the health facility.

1. Thank you again for agreeing to speak with us today, maybe you could begin by telling us a bit about yourself?
   - Family members and role in the family?
   - How long have you lived in this area?
   - How long have you been going to this facility?
   - Perception of health and illness
2. We would like to know your opinion about the primary health care services available in this area
   - Which facilities are available?
   - Any VHTs
   - Medical camps, campaigns etc.
3. When you or a member of your family gets unwell, who recognises and how do you decide what to do first?
   - Go to the facility
   - By drugs from the shop etc.
   - How is the decision made (who makes the decision and what factors are considered)
4. What about health information messages? Where do you get them? Which avenue is the most frequent?

Questions about facility experience

1. More specifically about the day that you visited the facility (where we met you before), which areas did you go through?
   - Registration
   - Clinician
   - Lab
   - Pharmacy
   - ANC
   - Maternity
   - Home visits
   - Any other areas that you have been to

Probe for each area:

- - 1. What was your experience like?
    2. Who did you interact with (relationship with health workers)?
    3. How were you received?
    4. Were you able to express yourself?- remember to not only ask about physical aspects of health or illness but also ask about psychological and emotional aspects of health and illness
    5. Did you have a good conversation with the musawo? – how was the treatment (Treatment process); were procedures explained, were you able to ask questions, were the questions answered. Are there questions that you wanted to ask but could not? Why?
    6. How did you feel at the end of it? (pay attention to patients’ feeling of involvement in decision making, confidence and patient enablement)

1. Why did you choose to go to that facility?
2. Are there things that you like about your experience at the facility?
3. Are there things that you don’t like about your experience at the facility?
4. If you could change something about the facility what would it be?
5. Are you aware of patient rights?
   1. If yes- what are they and where did you hear about them? If no, explain patient rights as written in the service charter
   2. What do you think about them?
   3. What is the situation with patients’ rights at the health facility that you visited (did you feel your rights were considered?)
6. What do you think are the responsibilities of a patient to a health facility? (ask about community ownership of the facility)
7. Is there anything else you would like to tell us about the facility or the health workers?
8. Do you have any questions for us?

**Thank the interviewee for their time**

**Tool: INTERVIEW GUIDE FOR FOCUS GROUP DISCUSSIONS WITH PATIENTS**

Okutegeera engeri edh’endhidandaba eyetololera ku mulwaire ku mutendera gw’amalwaliro agasookerwaku mu maserengeta ga eirungu lya Sahara-Ensonga ya Uganda

maserengeta ga Africa: Ensonga ya Uganda

*Understanding patient-centred care approaches at the level of primary health care facilities in sub-Saharan Africa: the case of Uganda*

**Akubagania asangaaza, yeyandula era n’okwandula kw’abenhigiremu**

**Facilitators welcome, introduction and introduction to participants**

Tusangaire era mwebale kwewayo kwenhigira mu luwayo luno olw’okukubagania ebidhuubo okw’aghalala. Endowoozayo yamugaso era tussima ebiseerabyo. Buti nandienze okuwa obubaka obusingawo ku luwayo lwaife olwa leero. (bita mu lupapula oluliku obubaka n’olusaba olukusa waigulu) ***Welcome*** *and thank you for volunteering to participate in this focus group. Your point of view is important and we appreciate your time. I would now like to give you some information about our discussion to day (go through the information sheet and consent process above).*

Buti nga buli omu bwali obulungi n’oluwayo, tulina obubaka obundi n’okusaba okundi:

*Now* *that everyone is comfortable with the discussion, we have some more information and some requests*:

- Kikulu okuba nti muntu mulala yenka n’ayogera ku kiseera, buli omu aidha kugheebwa omukisa okwogera. Wayinza okubaawo okusikirizibwa okubukiramu ng’omuntu alikwogera aye tusaba okulinda mpaka nga amaze.
- *It is important that only one person speaks at a time, everyone will be given an opportunity to speak. There may be temptation to jump in when someone is talking but we ask to wait until they have finished.*
- Wazira bidhuubo bituufu oba biffu, era tikikukakataku kwiikiriza nabandi mu kibindha.
- *There are no right or wrong opinions, and you don’t have to Ndikiririza with others in the group*
- Era timuteekwa kwogerera mu ntegeka yakwiiraganwaaku
- *you also don’t have to speak in any particular order*
- (Wa amawulire ku by’okunwa n’ebisenge awawumulirwa)…oli waidembe okufula muuku nga okubagania ebidhuubo kulikuja mu maiso aye tusaba nti okikole mpola
- *(Give information on refreshments and restrooms)…you are free to move out during the discussion but we ask that you do so quietly*
- Olinaku ebibuuzo byonabyona?
- *Do you have any questions?*
- Nkale, leka tutandike
- *Ok, lets begin*

**Twesuyemu**

***Warm up***

Okusookera irala nandienze buli omu yeyandule

*First I would like everyone to introduce themselves*

Ebigema ku muwi w’amawulire omukulu ku lupapula olw’amawulire (era kino kisobola okukolebwa aghalala n’okwandula)

Emyaaka, gha webaviira, eirwaliro lyebajaaku (fill in the demographic data capture form)

**Ekibuuzo ekitandika**

Nandienze okuwaayo edakiika ntono okulowooza ku bumanhirivubwo mu bifo eby’empeereza esookerwaku. Waliwo asanwiike okugabanaku niife kyaidhi?

***Introductory question***

*I would like to give you a few minutes to think about your experiences at primary health care facilities. Is anyone happy to share their experiences*?

Ebibuuzo ebitugalaga

Guiding questions

1. What do you think makes a facility of good quality?
2. Why do you choose to come to this facility?
3. As we begun, you said that one of the things that makes a facility good is _____

Is that available in Busowobi health centre?

1. If private facilities were also free, would you still come to this facility? And why?
2. Are there facilities that are not available at Busowobi health centre that you would like to be introduced?
3. For the services that you cannot get at busowobi, where else do you seek health care?
4. Are health workers available at the facility all the time? All the days of the week?
5. What do you do when the health workers are not available?
6. Now we are going to ask you questions about your experience at Busowobi health centre?
7. Before you go to the health facility, how or what things do you have to prepare?
8. What happens from the time you enter the facility to the time you leave?
9. Waiting time, patient flow, consultation, privacy and discussing personal problems or examination of sensitive areas? How do you feel about other patients being there?
10. What support do you get from the facility after you leave the facility?
11. Follow-up calls, if you get ill before the next appointment, if you have to do a test or get drugs from elsewhere…what happens?
12. What are some of the things that this facility does really well, or that you like about this facility?
13. What do you think can be improved and how? Any complaints or challenges?

Eriyo emirimu egyindi egy’omugaso oba ebikolebwa ebibairewo oba ebiriwwo ebirinawo kyebikoze ku mutindo gwempeereza efunibwa mu malwaliro agasookerwaku?

*Are there any other important activities or events that have happened or are happening that have an impact on the quality of care offered in the primary health care facilities?*

Ki kyewandisiinze kwenda kweega okubita mu mirimu gyaife?

*What would you be interested in learning through our work?*

Omuntu owundi gwetuyinza okwogeraku naye?

*Anyone else we should talk to?*

Wandienze okutegezebwaku ku bibindha mwetugya okunanooniaku okuwaya?

*Would you like to be informed of follow-up group discussions? if so kindly leave your contacts with us*

**Okumaliriza:** Webaze gw’olikubuuza olw’okwenhigiramu era omalirize okubuuza

***Conclusion****: Thank the interviewees for their participation and conclude the interview.*
